# Zinc finger oxidation of Fpg/Nei DNA glycosylases by 2-thioxanthine: biochemical and X-ray structural characterization

**DOI:** 10.1093/nar/gku613

**Published:** 2014-08-20

**Authors:** Artur Biela, Franck Coste, Françoise Culard, Martine Guerin, Stéphane Goffinont, Karola Gasteiger, Jarosław Cieśla, Alicja Winczura, Zygmunt Kazimierczuk, Didier Gasparutto, Thomas Carell, Barbara Tudek, Bertrand Castaing

**Affiliations:** 1 Centre de Biophysique Moléculaire, UPR4301, CNRS, rue Charles Sadron, 45100 Orléans cedex02, France; 2 Institute of Biochemistry and Biophysics PAS, Pawinskiego 5A, 02–106 Warsaw, Poland; 3 Department of Chemistry, Ludwig-Maximilians-Universität (LMU), Butenandtstr. 5–13 (Haus F), München D-81377, Germany; 4 Institute of Chemistry, Warsaw University of Life Sciences, 159C Nowoursynowska St., 02–787 Warsaw, Poland; 5 Laboratoire Lésions des Acides Nucléiques, SCIB/UMR E3 CEA-UJF, INAC, CEA, Grenoble, France; 6 Institute of Genetics and Biotechnology, Warsaw University, Pawinskiego 5a, 02–106 Warsaw, Poland

## Abstract

DNA glycosylases from the Fpg/Nei structural superfamily are base excision repair enzymes involved in the removal of a wide variety of mutagen and potentially lethal oxidized purines and pyrimidines. Although involved in genome stability, the recent discovery of synthetic lethal relationships between DNA glycosylases and other pathways highlights the potential of DNA glycosylase inhibitors for future medicinal chemistry development in cancer therapy. By combining biochemical and structural approaches, the physical target of 2-thioxanthine (2TX), an *uncompetitive* inhibitor of Fpg, was identified. 2TX interacts with the zinc finger (ZnF) DNA binding domain of the enzyme. This explains why the zincless *h*NEIL1 enzyme is resistant to 2TX. Crystal structures of the enzyme bound to DNA in the presence of 2TX demonstrate that the inhibitor chemically reacts with cysteine thiolates of ZnF and induces the loss of zinc. The molecular mechanism by which 2TX inhibits Fpg may be generalized to all prokaryote and eukaryote ZnF-containing Fpg/Nei-DNA glycosylases. Cell experiments show that 2TX can operate *in cellulo* on the human Fpg/Nei DNA glycosylases. The atomic elucidation of the determinants for the interaction of 2TX to Fpg provides the foundation for the future design and synthesis of new inhibitors with high efficiency and selectivity.

## INTRODUCTION

Physical and chemical agents from environmental or normal endogenous metabolism continuously damage DNA. To prevent the propagation and accumulation of mutations resulting from DNA damages, cells have evolved numerous DNA damage sensing and repair strategies which contribute to maintaining genome integrity and stability (1). Difficulties in repairing DNA damage may cause cellular dysfunction and death and can also potentially yield uncontrolled cell growth and cancer. Among repair strategies, the base excision repair (BER) pathway is the major line of defense against the deleterious effects of oxidized, alkylated and lost DNA bases (2,3). DNA glycosylases initiate the BER pathway by specifically recognizing and removing the base damage. Although these enzymes can be mono-functional by hydrolyzing the *N*-glycosidic bond using a water molecule (glycosylase) or bi-functional by using a reactive amine as nucleophile (glycosylase/lyase), they can be classified in six superfamilies according to their 3D folds, irrespective of their substrate specificities: the uracil-DNA glycosylase (UDG), the helix-hairpin-helix (HhH/GPD), the Fpg/Nei glycosylase, the alkyladenine-DNA glycosylase (AAG), the HEAT-like repeat (HLR) and the T4 endonuclease V structural superfamilies (4).

The Fpg/Nei superfamily (also called H2TH superfamily) includes bi-functional enzymes able to remove a wide variety of oxidized bases (3,5). The enzyme architecture consists in two globular domains connected by a flexible hinge: (i) a N-terminal domain rich in β-structures containing a highly conserved N-terminal α-helix (αA) displaying generally a proline (P1) at the N-terminal involved in catalysis and (ii) a C-terminal domain rich in α-structures containing both DNA binding domains of the enzyme (the H2TH motif and a β-hairpin domain structured in most cases as a Zinc finger, Supplementary Figure S1). All these enzymes feature AP lyase activity consisting in the successive cleavage at the 3’ and then at the 5’ sides of the abasic (AP) site according to β,δ-elimination. This yields a one nucleoside gap in DNA. After the action of 3’-phosphatase, the gap is filled in by a DNA polymerase and the resulting nick is sealed by a DNA ligase. Regarding substrate specificity, Fpg/Nei DNA glycosylases can be subdivided in two groups: (i) the Fpg proteins found in bacteria rather specific for oxidized purines such as 8-oxoguanine (8-oxoG) and the imidazole-ring opened purine (FapyG) and (ii) the Nei proteins found in bacteria and eukaryotes rather specific for oxidized pyrimidines in single- and double-stranded DNA such as thymine glycol (Tg), 5-hydroxycytosine (5OHC) and 5-hydroxy-5-methyhydantoin (Hyd) (6,7).

Although BER contributes to genome stability, there are synthetic lethal situations in which the inhibition of BER enzymes appeared a relevant strategy for cancer therapy. For example, PARP1 (a key BER/SSBR enzyme) inhibitors seem promising in a therapeutic approach to treating BRCA1/2- or PTEN-deficient tumors (deficiency in homologous recombination) (8,9). Otherwise, siRNA-mediated DNA glycosylase depletion in the osteosarcoma cell line (including the H2TH DNA glycosylase NEIL1) increases the cytotoxicity of chemotherapeutic agents (10). The functional depletion of NEIL1 also appears to be synthetic lethal with the depletion of Fanconi anemia DNA repair pathway (11). In another work, it has been shown that knockdown of the G:T mismatch-specific thymine-DNA glycosylase (MBD4) reverses methylation at specific *loci* which results in blocking breast cancer metastasis (12). Thus, selective inhibitors for MBD4 can be useful to prevent cancer metastasis. In a more recent study, Ramdzan *et al.* proposed a new mechanism to sustain proliferation in RAS-transformed cells through increased BER capability (13). In such a mechanism, the stimulation of the DNA glycosylase hOgg1 involved in the excision of the mutagenic 8-oxoG can be an alternative for RAS-transformed cells to overcome the antiproliferative effects of excessive oxidative DNA damage. These recent discoveries may provide new therapeutic windows in cancer therapy that could be exploited with selective drugs that specifically target DNA glycosylases.

**Figure 1.**
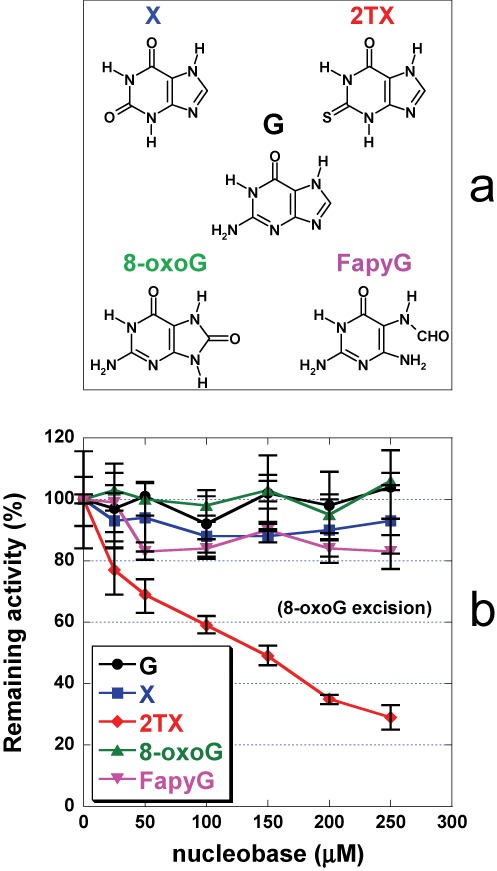
Inhibition of *Ll*Fpg by 2TX. **(a)** Structures of free nucleobases used in these experiments*.* Abbreviations are G for guanine, X for xanthine, 2TX for 2-thioxanthine, 8-oxoG for 7,8-dihydro-8-oxoguanine and FapyG for 2,6-diamino-4-hydroxy-5-formamidopyrimidine. **(b)** Effect of free nucleobases on Fpg 8-oxoG-DNA glycosylase activity. 25 nM of 24-mer 8-oxoG-DNA (Supplementary Table S1) and 5 nM of *Ll*Fpg were incubated 30 min at 37°C with 0, 25, 50, 100, 150, 200, 250 μM of G, X, 2TX, 8-oxoG or FapyG as indicated. Remaining activity (%) was plotted as a function of free nucleobase concentration. Each point corresponds to the mean value extracted from at least three independent experiments.

In a previous work, we initiated this research by exploiting the mechanism of the flip out of the damaged nucleoside-containing DNA and its extrahelical recognition inside the substrate binding pocket in an attempt to target the active site of the *Escherichia coli* Fpg protein (14). Because of the broad substrate specificity of Fpg, we screened 2,4,5,6-substituted pyrimidines and 2,6-substituted purines for their ability to inhibit the enzyme. 2-Thioxanthine (2TX, Figure 1a), one of the thiopurine analogues tested, prove to be the most efficient inhibitor of the excision of 2,6-diamino-4-hydroxy-5N-methyl-formamidopyrimidine (*N^7^*-Me-FapyG) by Fpg (14). Surprisingly, the inhibition mode of 2TX was determined as *uncompetitive* instead of the expected *competitive* mode. This suggested that 2TX binds to the enzyme/DNA complex outside the active site. By combining X-ray structure and functional studies on both Fpg and structural-related Fpg/Nei DNA glycosylases, we decipher at the atomic level the molecular basis of the mechanism by which the enzymes of this class are inhibited by 2TX.

## MATERIALS AND METHODS

### Chemical, proteins and DNAs

PAR reagent, guanine, xanthine were purchased from Sigma-Aldrich. 2TX was synthetized as described previously (14). The recombinant plasmid pFlag-CTC-WT-LlFpg encoding for the wt-*Ll*Fpg derived from the pFlag-CTC expression vector (15) and was used to generate single-point mutations W179A, R247G, C248GH and C268H. wt LlFpg and R247G and C168GH variants were overexpressed in *E. coli* BH540 (*fpg mutY*) strain and purified to homogeneity as already described for the wild-type enzyme. Other H2TH-DNA glycosylases were overexpressed with a C-terminal 6His-Tag in BL21(DE3) strain using the recombinant vectors derived from pET22b and pET30a for *Ec*Nei and *h*NEIL1, respectively (plasmids are gifts from Drs SusannWallace and Sylvie Doublié). *Ec*Nei and *h*NEIL1 were purified as already described (16,17). The 6His-hNEIL2 fusion protein was overproduced in *E. coli* BL21CodonsPlus cells using the expression vector pPR363 (a gift from Juan Pablo Radicella) and purified as previously described (18). Unmodified, THF- and 5OHC-containing single-stranded oligonucleotides were purchased from Eurogentec. Modified oligonucleotides containing Hyd and Bz-cFapyG were synthetized and purified as previously described (15,19,20). The structure of the damaged nucleosides and oligonucleotide sequences are reported in Supplementary Figure S2.

### Enzyme assays

For DNA binding experiments (electrophoresis mobility shift assay, EMSA) and DNA cleavage (glysosylase/lyase) assays, the damaged strands (containing either THF, Hyd, 8-oxoG or 5-OHC, Supplementary Figure S2) were 5’-[^32^P]-labeled before annealing with its complementary strand as previously described. Assays were performed in standard experimental conditions (21,22) except that all incubation mixtures contained 8% final concentration of dimethyl sulfoxide (DMSO) required for solubilizing the nucleobases (G, 8-oxoG, FapyG, X and 2TX, Supplementary Figure S1a). After electrophoresis, gels were exposed to autoradiography; scanned using STORM-Imager and quantified using ImageQuant software.

### Crystallization, X-ray diffraction data collection and structure determination

Protein/DNA complexes were obtained by mixing in a 1/1 molar ratio wt *Ll*Fpg, R247G or C248GH with a 14-mer DNA duplex containing either THF or Bz-cFapyG at a final concentration in protein of 2–5 mg/ml as already described (15,23,24). Crystals of R247G and C248GH bound to 14-mer THF-DNA were obtained in the conditions already described for the wt LlFpg. wt *Ll*Fpg/DNA complexes treated by 2TX were obtained by mixing one volume of the complex with one volume of a 2TX-saturated solution containing 0.1 M Hepes-NaOH, pH7.6, 1.2–1.4 M sodium citrate, 0.1–0.5% DMSO. The resulting drop was immediately equilibrated against the same solution by using the hanging drop vapor diffusion method. Usually, crystals appear after 2–3 days at 20°C and grow for 1–2 weeks.

Crystals were flash cooled in liquid nitrogen in the mother liquor. X-ray diffraction data were collected at 100K at beamline PROXIMA-1 (SOLEIL, Paris) or ID23–1 (ESRF, Grenoble). All data were processed using the XDS package (25) and the CCP4 program SCALA (26,27). The 3D structures were solved by molecular replacement using PHASER (28) and complexes previously reported by us (PDB code 1PM5 and 3C58) as search models. Atomic models were then refined using phenix.refine (29) and manually improved using COOT (30). Data collection and refinement statistics are listed in Supplementary Table S1. All structure figures were generated with CCP4MG (31).

### PAR assay

Essays were performed at room temperature in 200 μl final volume in 25 mM Hepes/KOH, pH7.5, 70 mM KCl, 5% glycerol, 8% DMSO containing 5 μM of enzyme (*Ll*Fpg, *Ec*Nei, *h*NEIL1, *h*NEL2 or available purified *Ll*Fpg mutant versions), 100 μM of 4-(2-pyridylazo)resorcinol (PAR) with or without 7.5 μM of 14-mer THF-containing DNA duplex and an excess of 10 mM of 2TX. In the presence of DNA, the mixture containing the enzyme, the PAR reagent and DNA were first pre-incubated at room temperature for 20 min to allow formation of the enzyme-DNA complex before 2TX addition. The release and re-sequestration of Zn ion was calorimetrically measured in the chelate form [Zn^2+^-PAR] at λmax 500 nm. Re-sequestration of Zn^2+^ by the PAR chelate was determined from a standard solution of ZnSO_4_. Data were presented as molar ratios of release (re-sequestered) Zn^2+^ to enzyme in percent. All titrations were performed in triplicate and data were averaged.

### Effect of 2TX and X on the 5OHC-DNA glycosylase activity of K21 cells

The Human fibroblast cell line K21 immortalized with hTERT (32) was a kind gift from Prof. M.H.K. Linskens, University of Groningen, The Netherlands. Cells were cultured in F10 Medium (+L-glutamine, Gibco), supplemented with 10% fetal bovine serum (Gibco) and grown at 37ºC in 5% CO_2_ in 100 mm cell culture dishes.

Cells at 90% confluence (3–4 ml cells per dish) were washed with 1× PBS and serum-free medium containing different doses of 2-thioxanthine (2TX) or xanthine (X) (0, 200, 500, 1000 μM). After 6 h of treatment, cells were washed with 1× PBS and then scraped into a 250 μl of cold Sonication Buffer (25 mM Tris-HCl, pH 7.5; 25 mM NaCl; 2 mM EDTA; 10% glycerol; 1× protease inhibitors cocktail – Roche). Next, cells were transferred to the ice chilled tubes and sonicated at the ‘High’ mode of the Bioruptor (Diagenode) sonicator. Three rounds of 15 s sonication and 30 s of break between the following pulses were done. Cell lysates were centrifuged (4000 *g*, 5 min) and the supernatants (cell-free extract) aliquoted and immediately frozen in liquid nitrogen and stored at –80°C. 5OHC-DNA glycosylase activity contained in the cell-free extracts was measured by using 5’-[^32^P]-34-mer 5OHC-DNA duplex as a substrate (Supplementary Figure S2). Note that 0.25 pmole of radiolabeled DNA probe was incubated for 1 h at 37°C with 20 μg of protein from the free K21 cell extracts in 30 μl in reaction buffer (25 mM Tris-HCl, pH 7.6; 25 mM NaCl; 2 mM ethylenediaminetetraacetic acid (EDTA)). Subsequently, proteins were digested for 1 h with Proteinase K (Sigma) (0.2 μg/μl of reaction mixture), and cleavage of the AP site generated by DNA glycosylases was performed by incubation with 0.2 N NaOH for 15 min at 65°C. Reaction mixtures were next neutralized with equimolar HCl and stopped by the addition of 25 μl of 95% formamide, 20 mM EDTA, 0.05% xylene cyanole and 0.05% bromophenol blue. DNA reaction products were then separated by 20% polyacrylamide gel (7M Urea) electrophoresis. Gels were exposed to a phosphoimager screen which subsequently was scanned. Radioactive bands were visualized with FujiFilm FLA7000 software. Intensities of bands were calculated with MultiGauge software.

### Viability of K21 cells after 2TX and X treatment

About 1000 cells were seeded to each well of 96-well plate and incubated for 16 h to enable cell adhesion. Then cells were washed with 1× PBS and serum-free medium containing different doses of 2TX and X in DMSO (0, 200, 500, 1000 μM) or DMSO alone was added. Cells were further incubated for 6 h at 37º C in 5% CO_2_ atmosphere. Subsequently, fresh medium with AlamarBlue was added and after further overnight incubation at 37º C in 5% CO_2_ fluorescence of living cells was measured at 540 nm/590 nm. Cell viability was calculated as a percent of untreated cells survival in the presence of DMSO alone.

### Mutagenesis experiments

The *in vivo* functionality of the *Ll*Fpg variants (W179A, R247G, C248GH and C268H) as compared to that of wt *Ll*Fpg was estimated by their abilities to complement the spontaneous mutator phenotype of the *E. coli fpg mutY* double mutant BH990 (derived from JM105). In practice, the frequency of rifampicine-resistant cells in 11 independent cultures was determined for BH990 cells expressing the wt or variants *Ll*Fpg proteins (33). Each protein was expressed in BH990 using the recombinant vectors pFlag-CTC (see above).

## RESULTS AND DISCUSSION

### Zinc finger of the Fpg protein is one of the targets of 2TX: evidence from crystal structures

The Fpg protein from *Lactococcus lactis* (*Ll*Fpg) (34) was our Fpg model for X-ray structure investigations. We first examined the effect of 2TX and other free purines on *Ll*Fpg 8-oxoG-DNA glycosylase activity (Figuer 1a). The excision of 8-oxoG from 24-mer DNA duplex (Supplementary Figure S2) by *Ll*Fpg was inhibited by increased 2TX concentrations (*red* curve, Figure 1b). The inhibition appears specific since the most canonical free nucleobase products of the enzyme (8-oxoG and FapyG) and the natural free nucleopurine (G) had no effect (*green*, *magenta* and *black* curves, respectively; Figure 1b). Although it is commonly accepted that damaged nucleobase products of Fpg do not inhibit enzyme activity, it has been shown that the free nucleotide 8-oxodGMP is able to inhibit *Ec*Fpg with *Ki* = 700 μM (35). Binding of 8-oxoG nucleoside inside the active site has been also reported in the crystal structure of Ogg1 from *Clostridium acetobutylicum* (36). The possible transient confinement of the free damaged nucleobase product within the active site has been already observed for other DNA glycosylases, such as hOgg1 (37). Thus, the free 8-oxoG base may be retained by hOgg1 (the human Fpg functional homologue) in the substrate recognition site after *N*-glycosidic bond cleavage. This results in retro-inhibition of the enzyme activity by decreasing its turnover and the lyase activity (38). Unambiguously, such inhibition by free 8-oxoG does not operate on the Fpg protein (*green* curve, Figure 1b). Interestingly, xanthine (X) which diverges from 2TX by only the nature of the substitution on position 2 (an oxo-group instead of a thione-group, Figure 1a), does not affect Fpg activity (*blue* curve, Figure 1b) suggesting that the thione-group of 2TX may be involved in the Fpg inhibition mechanism. The previous observation showing that other thio-nucleopurines and -pyrimidines have no effect on Fpg activity (14) suggests that the xanthine-moiety of 2TX combined with the 2-thione group jointly contribute to the inhibition. Overall, these data indicate that 2TX inhibits Fpg whatever its bacterial origin, *E. coli* or *L. lactis* ((14), Figure 1b).

**Figure 2.**
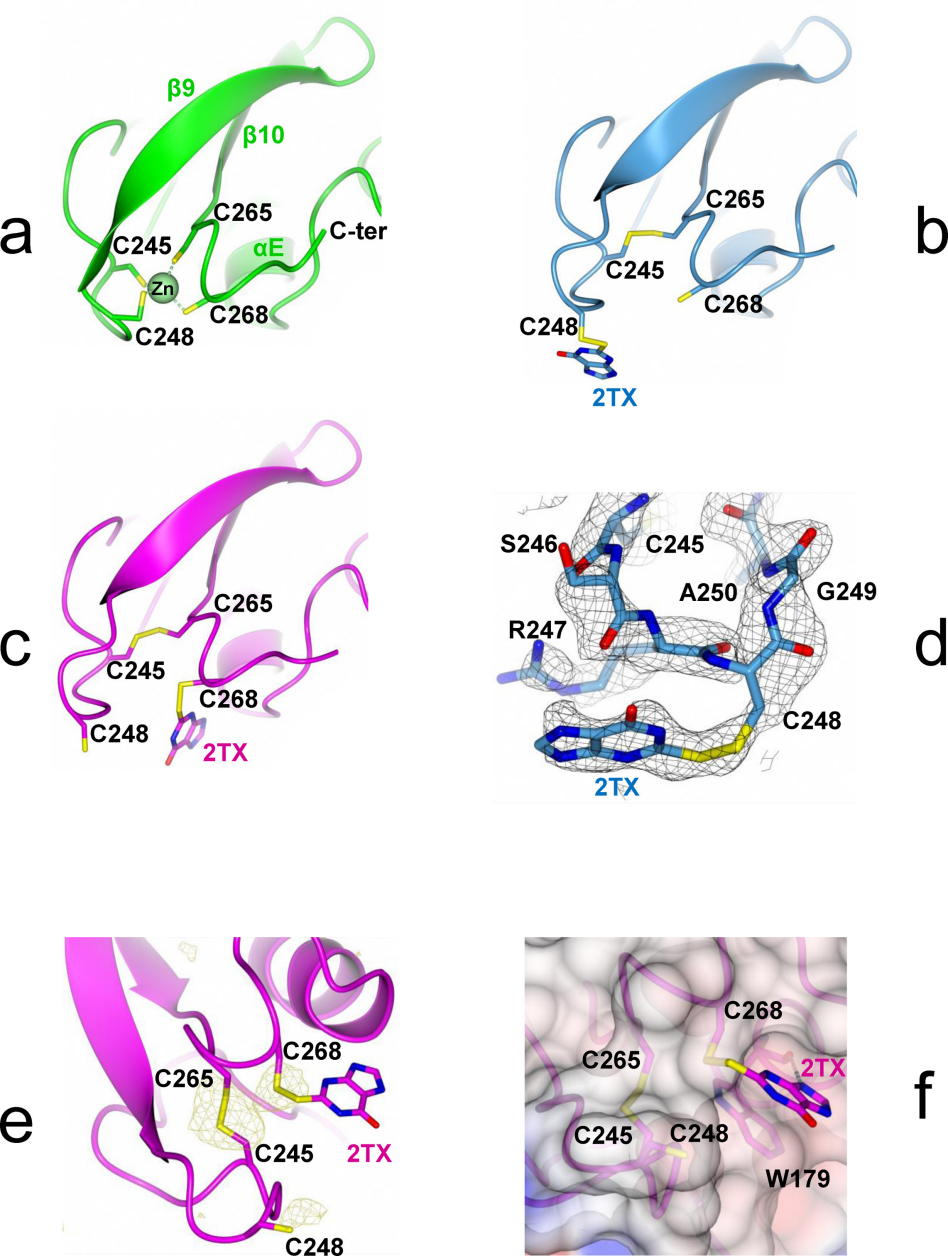
X-ray structures of Fpg zinc finger oxidized by 2TX. **(a)** Native Fpg zinc finger in the crystal structure of *Ll*Fpg bound to tetrahydrofurane(THF)-containing 14-mer DNA duplex (*green*, 1PM5). **(b)** and **(c)** Oxidized Fpg zinc finger in the crystal structure of *Ll*Fpg bound to THF- and Bz-cFapyG-containing DNA after treatment of the preformed protein/DNA complex with 2TX ([WT/THF]-2TX and [WT/Bz]-2TX in *blue* and *magenta*, respectively). **(d)** 2Fo-Fc Simulated Annealing omit map contoured at 0.7σ of [WT/THF]-2TX (in *black* grid). **(e)** Phased anomalous difference map contoured at 3.2σ of [WT/Bz]-2TX (in light *brown* grid). **(f)** View of the binding mode of 2TX to W179 in the crystal structure of [WT/Bz]-2TX. In all views, cysteine thiolates and disulfide bonds are indicated in yellow sticks. A superimposition of the structures in (a), (b) and (c) is shown in Supplementary Figure S3.

To elucidate the structural basis of the enzyme inhibition by 2TX, stable complexes of wt-*Ll*Fpg bound to substrate analog-containing DNA were crystallized in the presence of 2TX. We obtained crystals with 2TX-treated complexes of *Ll*Fpg bound to tetrahydrofurane (THF)- or the carbanucleoside of *N^7^*-Benzyl-FapyG (Bz-cFapyG)-containing 14-mer DNA duplexes ([WT/THF]-2TX and [WT/Bz]-2TX, respectively) (Supplementary Figure S2). The 3D structures of both complexes were solved by molecular replacement using PDB id codes 1PM5 ([WT-/THF]) and 3C58 ([WT/Bz]) as searching models and refined to 2.4 and 2.1 Å resolution, respectively (15,23) (Supplementary Table S1). Apart from the zinc finger domain of the enzyme (ZnF), the overall structures of *Ll*Fpg/DNA complexes treated by 2TX were very similar to each other and with starting models without drug (Figure 2). The common and most striking feature of both structures obtained with 2TX was the absence of the zinc ion which is associated with a conformational change in the zinc coordination sphere residues (ZnF cysteines and the peptide S246-A250). In both cases, the tetrahedral zinc ion initially coordinated by four cysteines in 1PM5 and 3C58 (C245, C248, C265 and C268, Figure 2a) was no longer present (Figure 2b and c). Moreover, most buried cysteines of Fpg-ZnF (Supplementary Table S2), Cys245 and Cys265, were covalently linked together by forming a disulphide bridge.

In the structure of [WT/THF]-2TX, C268 kept the same conformation as the one observed in the structure of [WT/THF]. The associated partial structural change from the N-terminal side of ZnF (the peptide 246–250 including part of the zinc coordination sphere residues and the start of the β9-strand, Supplementary Figure S1c) results in exposing the C248 side chain to the solvent as seen in the increase of accessible surface area from 3.1 Å^2^ in 1PM5 to 21.4 Å^2^ in the structure with 2TX (Figure 2a and b, Supplementary Table S2) (sulfur accessible surface areas were calculated with POPS, (39)). Additional density near the S atom of C248 suggested that a 2TX molecule was bound to the enzyme by a disulfide bond. A 2TX molecule was then modeled in SigmaA-weighted 2mfo-Dfc electron density maps. Following refinement of the structure of [WT/THF]-2TX (Supplementary Table S1), the average B-factor of the covalently linked 2TX molecule was 88.9Å^2^ which may result from partial occupancy and/or multiple conformations (Figure 2b and d). In addition, some extra density was seen near the S atom of C268 and might be attributed to the S atom of a second 2TX molecule bound to this cysteine by a disulfide bond. Electron density for the other atoms of the 2TX xanthine-moiety was extremely weak and refinement statistics with a second 2TX molecule bound to Fpg did not lead to satisfying values. This is certainly due to very low occupancy and multiple conformations. Therefore, a 2TX molecule (potentially bound to C268) was omitted from the last refined model.

Contrary to the structure of [WT/THF]-2TX, a 2TX molecule covalently bound to C268 was easily modeled in the electron density map of the crystal structure of [WT/Bz]-2TX (Figure 2c). In addition, an X-ray diffraction data set was collected at a wavelength of 1.55Å to exploit the anomalous signal of S atoms (Supplementary Table S1). The phased anomalous density map shows unambiguously the position of S atoms of the modified ‘zincless’ finger (the disulfide bonds between C245 and C265 and between C268 and 2TX, Figure 2e). Some extra density was also seen near the S atom of C248 and might be attributed to the S atom of a 2TX molecule bound to this cysteine by a disulfide bond (but it was omitted from the last refined model). Interestingly, the xanthine-moiety of the 2TX molecule bound to C268 makes π-π stacking interactions with the aromatic ring of W179 and an H-bond from its N(9) to the carbonyl O atom of the main chain of the same Fpg residue (Figure 2f).

Structural studies of Fpg/DNA complexes treated by 2TX indicated that 2TX is able to oxidize the zinc finger of the enzyme (Figure 2 and Supplementary Figure S3). One of the Fpg targets of 2TX is localized outside the active site and thus illustrating the marked *uncompetitive* component of the inhibition (14). Interestingly, the binding of 2TX to W179 and its covalent link to C245 or C268 are also in good agreement with single-turnover inhibition experiments which suggested that the thione function and the xanthine ring-moiety of 2TX are both needed for inhibition (Figure 1). The stacking between the π-orbitals of 2TX and W179 requires the imidazole ring of 2TX and thus can explain why the thio-pyrimidines tested previously were unable to inhibit the enzyme (14). In addition, the H-bond between N(9) of the imidazole ring of 2TX and the CO backbone of W179 observed in the crystal structure suggests that the N(9) of 2TX is protonated. The N(9)H tautomer of 2TX is known to co-exist in solution with the N(7)H tautomer and it was proposed to be the active form for the inhibition of the *E. coli* Fpg protein (14,40–42). 3D structures revealed that 2TX is able to chemically react with the two most exposed cysteines C248 and C268 of Fpg-ZnF (Supplementary Table S2), resulting in their oxidation and the loss of the Zn ion. These structural observations highlight a possible irreversible inhibition process associated with the loss of Zn^2+^ in solution.

### 2TX reacts in solution with both the free and DNA-bound Fpg protein inducing zinc release and abolishing DNA binding

**Figure 3.**
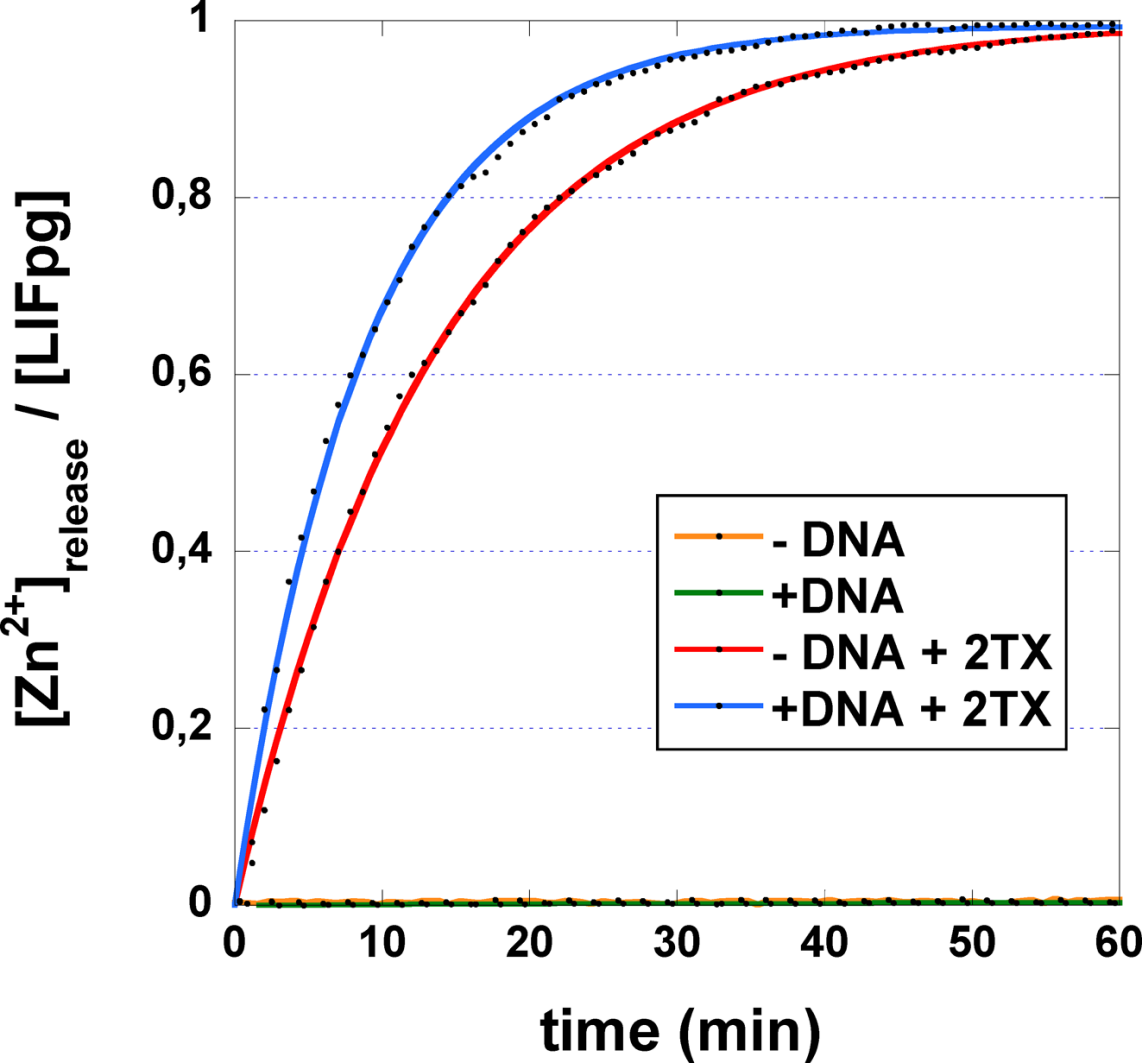
Time course of 2TX-induced Zn-release from the free and DNA bound *Ll*Fpg. *Ll*Fpg or the preformed *Ll*Fpg/14-mer THF-DNA complex (Supplementary Figure S2) was incubated at room temperature with (*red* and *blue* curves, respectively) or without (*orange* and *green* curves, respectively) 2TX in the presence of PAR. The release of Zn^2+^ ion contained in *Ll*Fpg induced by 2TX is followed by its re-sequestration by the PAR reagent which absorbs at 500 nm under its chelated form [PAR]_2_-Zn^2+^. Data (*black* points) corresponding to the mean values of three independent kinetics were plotted as a function of time and, when Zn2+ was released, fitted by applying [Zn^2+^]_release_/[*Ll*Fpg] = A_0_. (1-exp(-*k_obs_*.t)), where A_0_ is the total [Zn^2+^] extracted from *Ll*Fpg (i.e. [Zn^2+^]_release_/[*Ll*Fpg] = 1), *k_obs_* the observed first-order kinetic constant and *t* the incubation time.

The common structural feature revealed by crystal structures of Fpg/DNA complexes treated by 2TX is the oxidation of the enzyme zinc finger (ZnF) associated with the loss of the zinc ion. To eliminate any bias due to the crystallization medium and/or a special chemistry related to the peculiar molecule arrangement in the crystal, it was necessary to examine the Zn ion extrusion in solution. There is a direct method for assaying the presence of micromolar concentrations of free Zn(II) ions in solution. This method exploits differences in the visible absorption spectra of the chromophoric chelator 4-(2-pyridylazo)resorcinol (PAR) after its complexation with Zn^2+^ (43). We used the PAR assay to verify if 2TX can induce the loss of the Zn^2+^ ion in solution as suggested by X-ray studies. To examine how DNA modulates the action of 2TX, the experiments were performed in the presence or in the absence of THF-containing 14-mer DNA duplex (Supplementary Figure S2). For that *Ll*Fpg alone or bound to THF-DNA were incubated in the presence of 2TX and PAR reagent. The formation of the PAR- Zn^2+^ chelate, attesting the presence of free Zn^2+^ in solution, was followed as function of time by its absorption at 500 nm (Figure 3). 2TX is able to extract the Zn ion in solution from the enzyme bound to DNA (*blue* curve, Figure 3). Interestingly, this experiment also revealed that 2TX induces Zn loss from the free enzyme (*red* curve, Figure 3). This suggests that a *non-competitive* inhibition mode mediated by 2TX could contribute to the inhibition. In this case, the inhibitor binds outside the active site and has an equal affinity for the enzyme and the enzyme-substrate binary complex. The difficulty of detecting a possible *non-competitive* inhibition mode in kinetic experiments (14) can be explained by assuming that the binding of the enzyme to its DNA substrate is much faster than the binding of 2TX to the enzyme. Consequently in the presence of DNA, 2TX essentially encounters in solution the enzyme bound to its DNA substrate. In such a situation, it is the *uncompetitive* inhibition mode which essentially contributes to the global inhibition observed. Although in the same range of magnitude, the DNA-bound enzyme appears slightly more sensitive to 2TX (almost 1.6 times) than the free enzyme as indicated by the apparent Zn-release kinetic constant, *k_obs_* (Table 1). Such a situation indicates that the inhibitor may bind the free enzyme and the enzyme-substrate complex differently. We have no structural evidence to explain why the enzyme bound to DNA is more sensitive to 2TX. Indeed, the Zn-Cys coordination core of Fpg ZnF appears similarly accessible in both situations. With the exception of some flexible loops of the enzyme which locally rearrange through binding, the global fold of the free and bound enzyme does not change significantly, especially the ZnF core (24). Probably, the presence of DNA creates a better chemical environment for 2TX. We surmise that the binding of 2TX is similar in both situations but that its reactivity against ZnF cysteine thiolates is increased by the presence of DNA.

**Table 1.** Observed kinetic constants for 2TX-induced Zn release of ZnF-containing H2TH DNA glycosylases

Proteins		*k_obs_* (min^-1^)^a^		*k_obs_* ratio^b^
		- DNA	+ DNA^c^	
*Ll*Fpg	WT	0.073 ± 0.001	0.113 ± 0.004	1.55
	R247G	0.212 ± 0.005	0.333 ± 0.004	1.57
	C248GH	0.045 ± 0.001	0.075 ± 0.001	1.67
*Ec*Nei	WT	0.296 ± 0.001	0.452 ± 0.002	1.53
*h*NEIL2	WT	0.180 ± 0.002	0.299 ± 0.003	1.66

a Mean values of three independent experiments (±SD). b *k_obs_* ratio = *k_obs_*^(+DNA)^/*k_obs_*^(-DNA)^. c 14-mer THF-DNA (Supplementary Figure S2). See also the caption of Figure 3.

**Figure 4.**
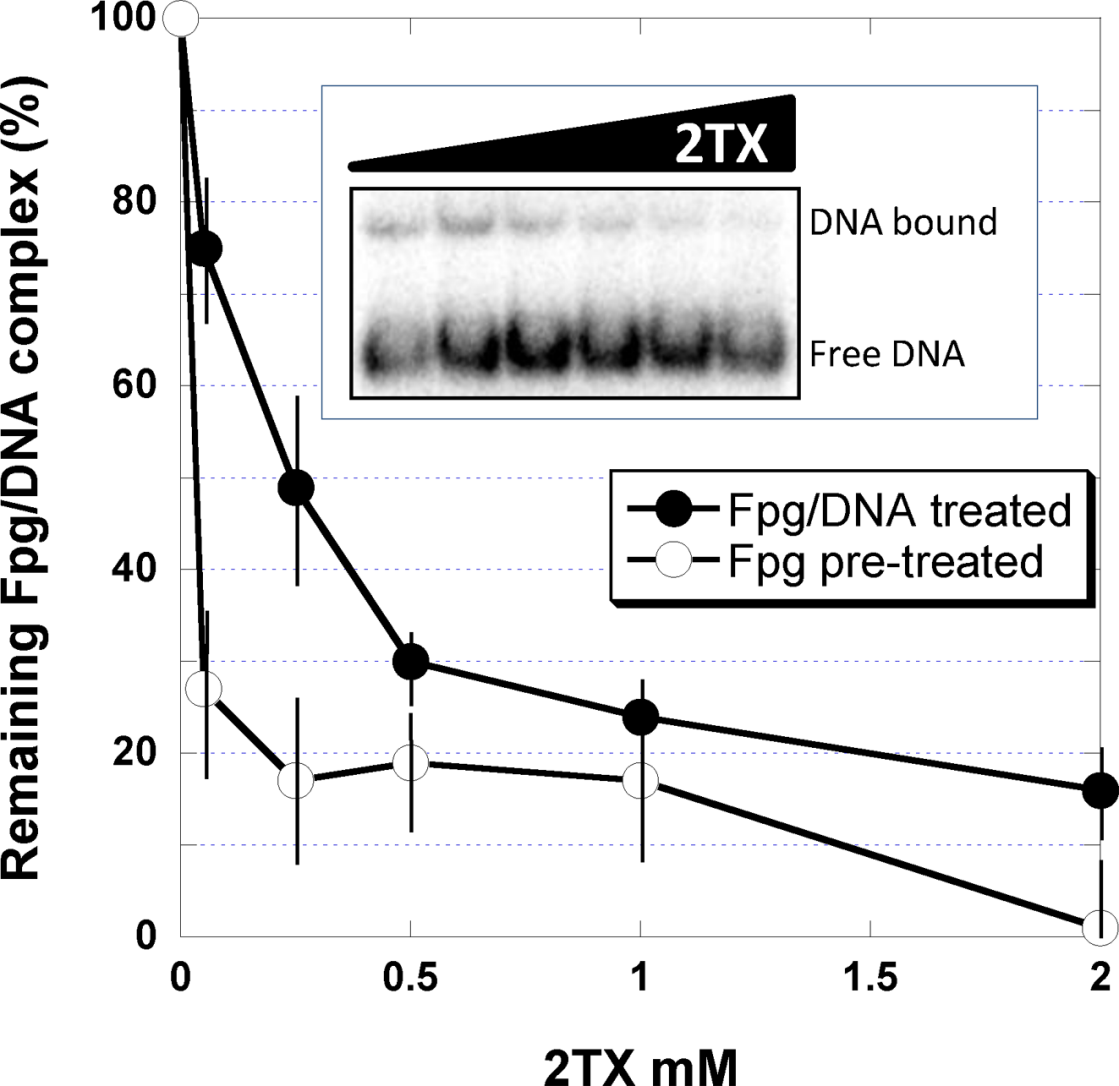
2TX-induced inhibition of *Ll*Fpg DNA binding and *Ll*Fpg/DNA dissociation. Protein/DNA complex was analyzed by EMSA (see Supplementary Figure S4) using as DNA probe the 14-mer THF-DNA (Supplementary Figure S2). Binding experiments were carried out with 2 nM *Ll*Fpg pre-incubated on ice for 20 min with indicated 2TX concentrations. After that, DNA was added to the pre-incubation mixture and incubated for 20 min on ice before EMSA analysis (*open* circles). The stability of the Fpg/DNA complex was studied as follows: the enzyme (2 nM) and DNA (0.1 nM) were first pre-incubated for 20 min on ice. The resulting pre-formed protein/DNA complex was then incubated for an additional 20 min on ice with indicated 2TX concentrations (*filled* circles). An example of such an experiment is shown in the insert. Data were normalized to the Fpg/DNA complex observed in the absence of 2TX (about 20–25%, see insert and Supplementary Figure S4). Each experimental point represents the mean value from three independent experiments.

Fpg ZnF being one of the DNA binding domains of the enzyme (H2TH domain, the second one, Supplementary Figure S1), it can be expected that in solution its oxidation by 2TX will significantly impair the enzyme DNA binding. To provide new insight into this problem, we analyzed the effect of 2TX on the ability of Fpg to bind specifically to damaged DNAs using an electromobility shift assay (EMSA). We exploited here the possibility of the enzyme to form abortive and stable complexes with substrate analogue-containing DNAs that we had previously designed and synthetized for solving the X-ray structures of Fpg bound to DNA. THF was used as an abasic (AP) site analogue (Supplementary Figure S2). THF-DNA duplex is recognized with a high affinity by *Ll*Fpg while being unable to process them (15,19,44). At first, the effect of 8% DMSO (used to solubilize 2TX) was examined on the titration of 14-mer THF-DNA by *Ll*Fpg (Supplementary Figure S4). Under the condition used, the concentration of enzyme needed for half-maximal binding is equal to the apparent dissociation constant (*K_D_app*) of the protein/DNA complex considered (44). Although in the same range of magnitude, the presence of DMSO in the reaction mixtures decreased 3 times *K_D_app* determined for THF-DNA. This means that DMSO stabilized the enzyme/DNA complex and/or stimulated the binding property of the enzyme. Such an effect of DMSO on the stability of nucleoprotein complexes has been already observed and is known to counteract the dissociation effect of salt (45). The effect of increased 2TX concentration (in 8% DMSO) on DNA binding activity of *Ll*Fpg and the stability of *Ll*Fpg/DNA complexes was next analyzed (Figure 4). In the same concentration range, 2TX strongly inhibited the DNA binding activity of the enzyme (*open* circle, Figure 4) and induced the dissociation of the preformed Fpg/DNA complex (*filled* circles, Figure 4). As observed above using the PAR assay, this means that both the free and the bound enzyme are exposed to the deleterious effect of 2TX. This also suggests that, in solution, the disruption of zinc coordination in Fpg ZnF by 2TX impairs the DNA binding of the enzyme and consequently the enzyme activity. This observation was not surprising since it has long been known that the Fpg ZnF integrity is essential for enzyme binding to its DNA substrate. Indeed, site-directed mutagenesis of cysteines belonging to *E. coli* Fpg ZnF (resulting in point mutations Cys to Gly, Ala, Ser or His) abolishes the enzyme DNA binding (46–48). In addition, Cys to Gly mutations affecting Cys residues outside ZnF do not impair catalytic and DNA binding properties (46,47). The effect of 2TX also compares well with the observation that non-cytotoxic concentrations of cadmium (Cd(II)), nickel (Cu(II)) and mercury (Hg(II)) inhibit Fpg (49). Because all these metals are known to be exchangeable with Zn(II) of Fpg ZnF (47), it has been proposed that metal inhibition results in disturbing the native structure of Fpg ZnF and consequently the Fpg DNA binding and catalysis. Surprisingly, 2TX-induced oxidation of Fpg-ZnF did not significantly affect, in the crystal, the β-hairpin structure (β9-β10) of the DNA binding domain (Supplementary Figure S1). Especially, the invariant Arg260 residue of the β9-β10 loop maintains its interactions with both DNA backbone phosphates bordering the flipped out damaged nucleoside (15,24). The compactness of the resulting ‘zincless’ finger domain observed in crystal structures is probably maintained partly by the disulfide bridge Cys245-S-S-Cys265 (Figure 2) and mainly by the crystal packing interactions which prevent the domain from locally unfolding.

### 2TX is a general inhibitor of zinc finger-containing Fpg/Nei DNA glycosylases

**Figure 5.**
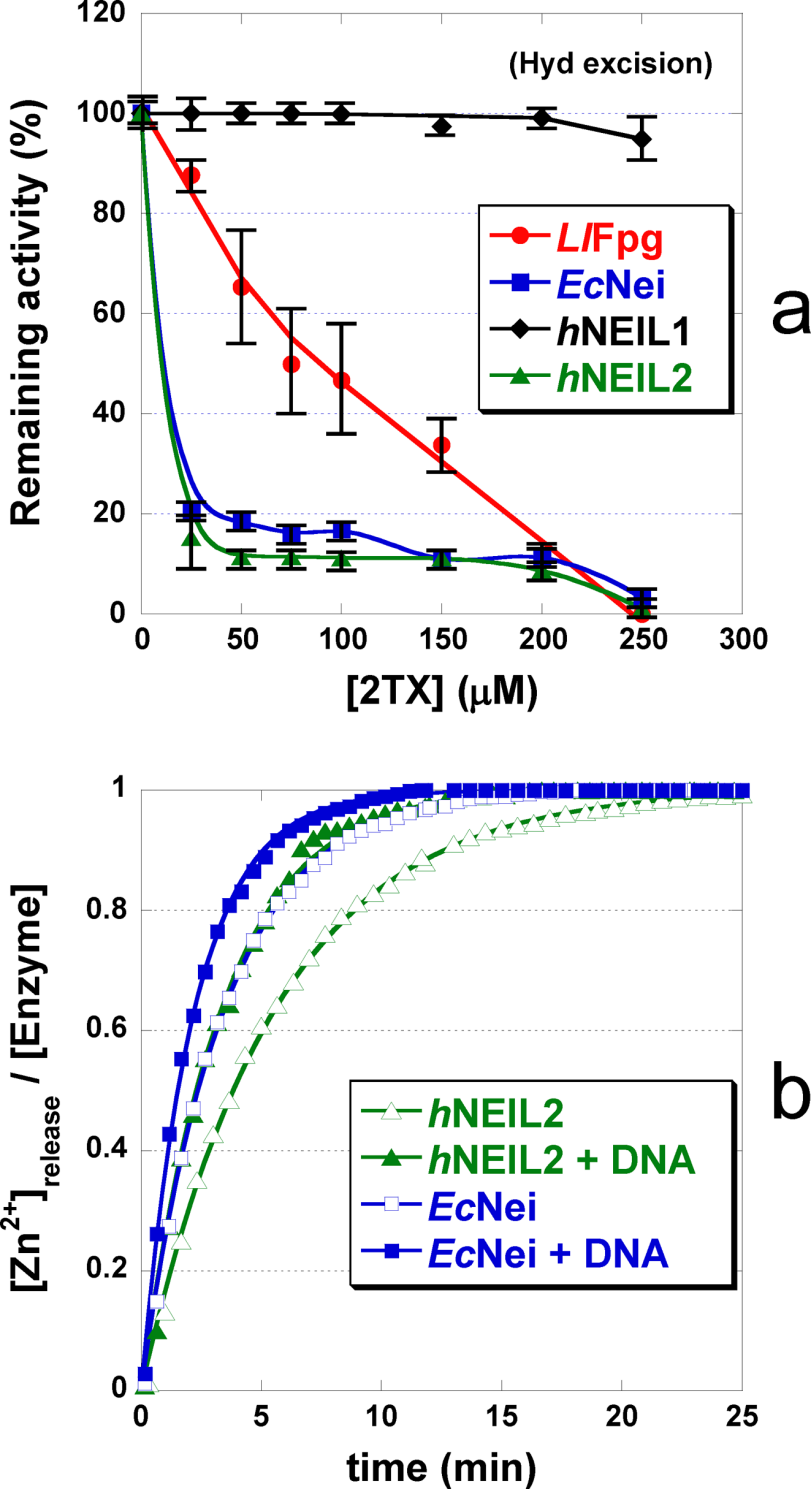
2TX-induced inhibition of zinc finger-containing Fpg/Nei DNA glycosylases is associated with the loss of Zn^2+^ ion. **(a)***Inhibition of Fpg/Nei Hyd-DNA glycosylase activity by 2TX* 1 nM of single- (with 10 μM *h*NEIL2) or double-stranded 5’-[γ^32^P]-24-mer oligonucleotide containing 5-hydroxy-5-methylhydantoin (Hyd, Supplementary Figure S2) opposite G (with 7 μM *Ec*Nei and 8 μM *h*NEIL1) or C (with 5 μM *Ll*Fpg) were incubated 30 min at 37°C in the presence of increased concentrations of 2TX. Incubation mixtures were then analyzed by Urea-PAGE and quantified as described in Materials and Methods section. **(b)***Time-course of 2TX-induced Zn-release of EcNei and hNEIL2* PAR assays were carried out as described in *Materials and Methods* section under the same conditions used in experiments presented in Figure 3. DNA corresponds to single-stranded 24-mer Hyd and double-stranded 24-mer Hyd:G-DNA for *h*NEIL2 and *Ec*Nei, respectively (Supplementary Figure S2).

The inhibition of Fpg by 2TX led us to explore the ability of this drug to inhibit other Fpg-related enzymes from the Fpg/Nei structural superfamily. In this aim, we analyzed the effect of 2TX on the excision of the 5-hydoxy-5-methylhydantoine (Hyd)-containing 24-mer DNA duplex by *Ll*Fpg, the *E. coli* Nei protein (*Ec*Nei) and the human Nei-like proteins 1 and 2 (*h*NEIL1 and *h*NEIL2) (see Supplementary Figure S2 for the structure of Hyd). As previously shown, Hyd-containing single-stranded DNA, double-stranded DNA with Hyd opposite C and G is a substrate for *h*NEIL2, *Ec*/*Ll*Fpg and *Ec*Nei/*h*NEIL1, respectively (22,50). Assays were performed in the presence of increased concentrations of 2TX (Figure 5a). The Hyd-DNA glycosylase of *Ll*Fpg was inhibited by 2TX (*red* curve, Figure 5a) as were the *N^7^*-Me-FapyG- (14) and 8-oxoG-DNA glycosylases (*red* curve, Figure 1c). This indicates that 2TX inhibits the enzyme whatever the nature of the damaged base-containing DNA substrate considered (oxidized purines or pyrimidines). The Hyd-DNA glycosylase activity of *Ec*Nei and *h*NEIL2 were also strongly inhibited by 2TX (*blue* and *green* curves, respectively, Figure 5a). As expected, 2TX did not affect the Hyd-excision by the *h*NEIL1 protein which contains a finger motif devoid of zinc (*black* curve, Figure 5a) (51). This reinforces the structural observations suggesting that ZnF of these enzymes is the target for 2TX (see below). As above, we estimated the specificity of 2TX for Fpg/Nei-DNA glycosylases by comparing its effect on Hyd-DNA glycosylase with those of other free nucleobases (Supplementary Figure S5). For all the enzymes considered, G, X, FapyG and 8-oxoG nucleobases had no significant effect on Hyd-DNA glycosylase (a weak inhibition by FapyG was observed for *Ec*Nei and *h*NEIL2). Comparison between the effect of X and 2TX confirms again that the thio-function of 2TX plays an essential role in the enzyme inhibition as suggested above for the inhibition of the Fpg 8-oxoG-DNA glycosylase (Figure 1c).

The singular character of *h*NEIL1 resistance against 2TX inhibition immediately suggests that the binding and reactive site(s) (i.e. ZnF) for 2TX leading to enzyme inhibition is absent in this protein. Crystal structures of free *h*NEIL1 and the Mimivirus NEIL1 (*mv*Nei1) bound to DNA reveal that the global folds of these proteins are very similar to those of bacterial Fpg/Nei proteins, *mv*Nei2 and human *h*NEIL3 (51–55). The peculiarity of NEIL1 orthologs relies on the absence of ZnF (a structural motif present in most of the other Fpg/Nei DNA Glycosylases) which is replaced by a ‘zincless finger’, ZnLF. Both ZnF and ZnLF domains of Fpg/Nei DNA glycosylases are superimposable especially in the β-hairpin motif level and play the same role in DNA binding (Supplementary Figure S1). Combining all these structural and biochemical data, it is reasonable to conclude that ZnF of Fpg/Nei DNA Glycosylases is a general target for 2TX. However, with the exception of *Ll*Fpg which contains only 4 cysteines all involved in Zn coordination, the other Fpg/Nei DNA glycosylases (including *Ec*Fpg) have additional free cysteines in their primary structures which could be potentially capable of reacting with 2TX. If 2TX is able to react with these free cysteines, this does not result in enzyme inhibition in particular for *h*NEIL1. For *Ec*Nei and *h*NEIL2, it was however necessary to determine whether 2TX is able to induce the release of the zinc ion from ZnF in order to confirm the inhibition mechanism. Unambiguously, the incubation of 2TX with *Ec*Nei and *h*NEIL2 results in the loss of Zn (Figure 5b). As for Fpg, the zinc loss is observed for the protein alone and for the protein/DNA complexes. The apparent kinetic constants for the release/re-sequestration of Zn(II) are greater when *Ec*Nei and *h*NEIL2 are bound to DNA (about 1.6 times, Table 1). Although it was difficult to fully assess whether one enzyme is more sensitive than the other, 2TX-induced zinc extraction appears more effective on *Ec*Nei and *h*NEIL2 than on *Ll*Fpg (about 4 and 2.5 times, respectively, Table 1). Interestingly, 2TX also inhibits *Ec*Nei and *h*NEIL2 more effectively than *Ll*Fpg in the same 2TX-concentration range (Figure 5a). Thus, there is a direct relationship between the inhibition power of 2TX and its ability to induce the loss of zinc (i.e. ZnF oxidation).

**Figure 6.**
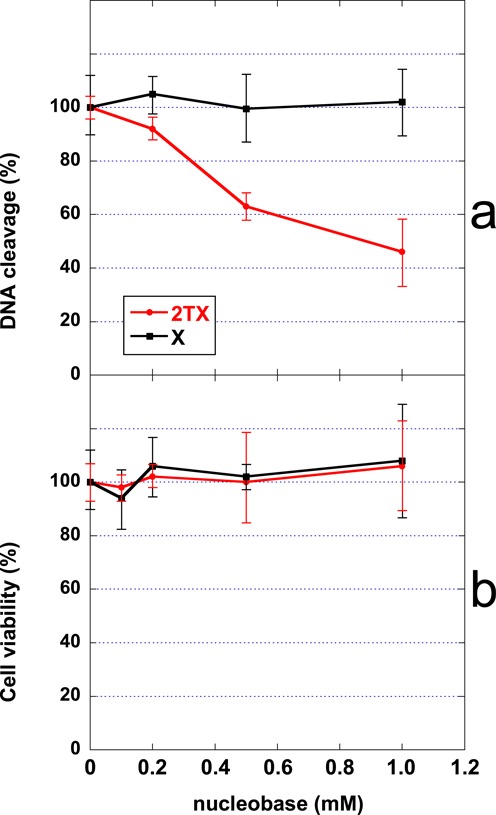
Effect of 2TX *in cellulo*. **(a)** Effect of 2TX and X on the 5-OHC-DNA glycosylase activity. **(b)** Effect of 2TX and X on cell survival. Human fibroblast K21 cells were treated for 6 h with indicated concentrations of 2-thioxanthine (2TX) or xanthine (X). Relative 5-OHC-DNA glycosylase activity in cell-free extract and cell viability were determined and plotted as a function of the nucleobase concentration used. Each point corresponds to the mean value of three independent experiments.

In order to investigate in living cells the possible effect of 2TX on the repair activity of the human Fpg/Nei DNA glycosylases, human fibroblasts from the K12 cell line were treated for 6 h either with 2TX or its analogue xanthine (X), which did not inhibit repair enzymes *in vitro* (Figure 1a). The 5-OHC (5-hydroxycytosine)-DNA glycosylase partly associated with *h*NEIL2 was measured in cell extracts after treatment. Incubation of cells with 2TX decreased in a dose-dependent manner the 5-OHC excision from DNA by cell-free extracts while X had no effect (red and black curves, respectively, Figure 6a). Such a decrease of repair was not associated with 2TX toxicity, since none of the concentrations of 2TX or X used inhibited cell viability (Figure 6b). These data suggest that 2TX may also inhibit 5-OHC-DNA glycosylase activity of *h*NEIL2 in living cells without inducing cell death. The inhibition effect of 2TX appeared incomplete and reached around 50%. This partial inhibition was not surprising since DNA glycosylases such as *h*NTH1 (from the HhH superfamily) and *h*NEIL1 (insensitive to 2TX *in vitro*, Figure 5a) have overlapping substrate specificities and serve as backups for each other (56).

### Proposed mechanism for the inhibition of ZnF Fpg/Nei-DNA glycosylases by 2TX

**Figure 7.**
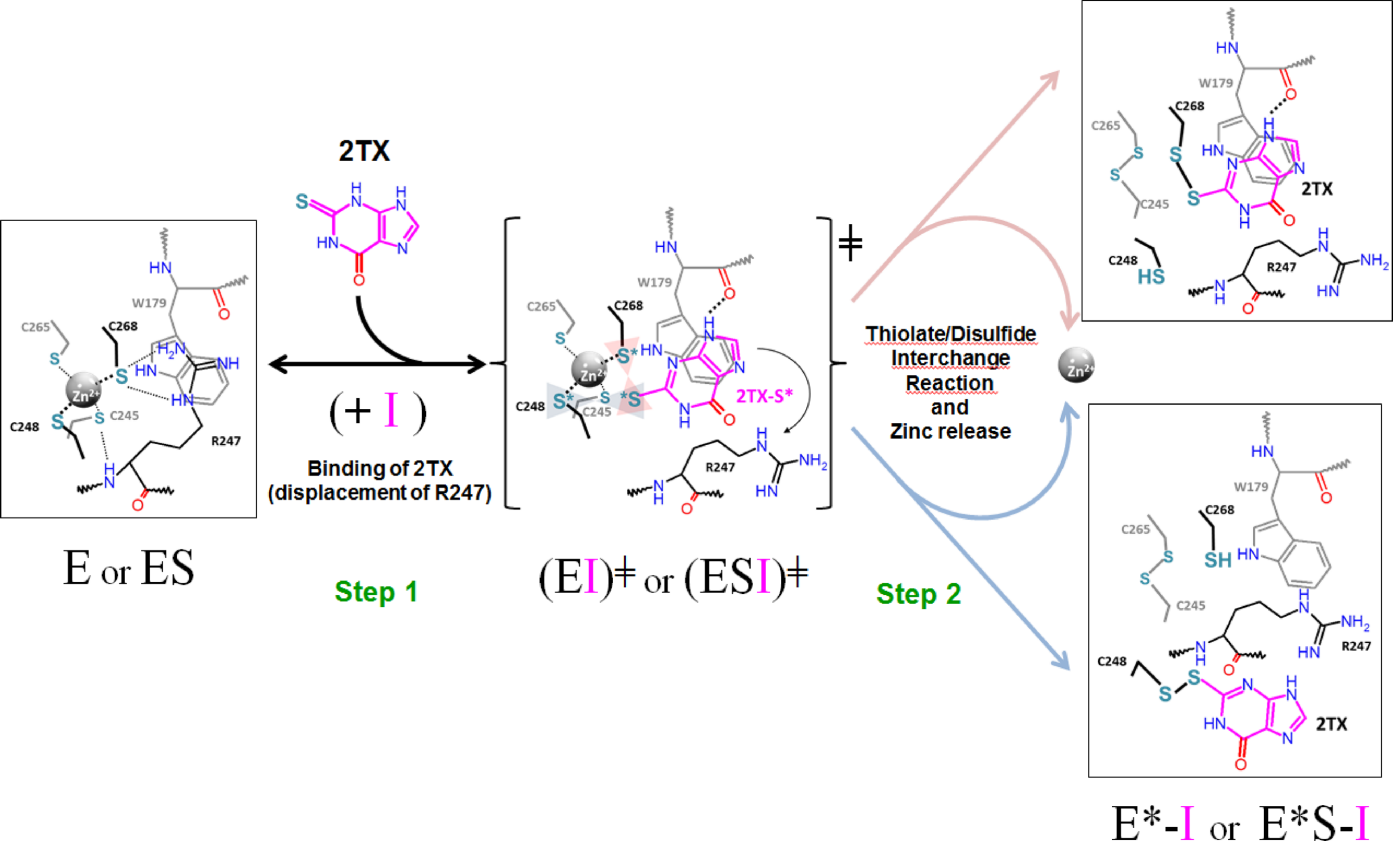
Molecular mechanism for 2TX-induced Fpg ZnF oxidation. Although kinetic inhibition of the enzyme (E) revealed that 2TX (I, in *magenta*) acts mostly as an *uncompetitive* inhibitor (binding of the inhibitor to the enzyme/substrate complex, ES), PAR assay and EMSA unambiguously demonstrated that 2TX is able to induce the release of Zn^2+^ ion from the free enzyme (a *non-competitive* inhibition behavior) (see text). Thus, the proposed molecular mechanism assumes that in a first step, (I) can bind both E and ES to form EI or ESI transient complexes. Through specific binding (π-orbital stacking and one H-bond formation with W179), 2TX displaces R247 which results in the activation of C248 and C268 thiolates and its thione-function activation (S*, in *cyan*). The transient complex (EI^╪^ and ESI^╪^), readily and irreversibly leads to the inactivated enzyme complex (E*-I and E*S-I) in which the enzyme has lost its Zn^2+^ ion (E*). Only the structures of (E*S-I) observed in the crystal structures of *Ll*Fpg/DNA complex-treated by 2TX are shown schematically in the figure. For more details, see the text.

The present data suggest that the inhibition of Fpg/Nei-DNA glycosylases by 2TX can result from two successive molecular events: the reversible binding of the thiopurine to the enzyme and the chemical reaction leading to (i) the covalent linking of 2TX to C268 or C248 thiolates and (ii) the Zn loss. Crystal structures of Fpg/DNA complexes in the presence of 2TX showed unambiguously that the target site of 2TX is localized outside the active site at the interface between the H2TH and ZnF DNA binding domains of the enzyme. It can be subdivided into at least one binding site and two reactive sites: W179 belonging to the helix αE of the H2TH motif and the most exposed C248/C268 of ZnF, respectively (Figure 2, Supplementary Figure S3). Thus, the molecular mechanism for 2TX-mediated *Ll*Fpg inhibition can be described by two steps: (step 1) 2TX selectively binds to W179 and (step 2) reacts with C248 or C268 thiolates which results in the Zn-coordination sphere destabilization associated with the Zn release. As suggested by structural and biochemical studies, this two-step mechanism can operate on the free (E) and DNA bound enzyme (ES) (Figure 7).

Considering the polar character of 2TX (associated with a strong negative electrostatic potential around the thione function, (42)), the positively charged surface of the Zn-coordination sphere (defined by the basic residues R247 and K182) is clearly favorable to the approach and to the electrostatic interaction of 2TX to its binding site. The stabilization of the interaction relies next on the π-orbital stacking between the imidazole ring of 2TX and the indole ring of W179 reinforced by the H-bond between the N(9)H of 2TX and the carbonyl of W179 (Figure 2f). Through binding to W179, 2TX replaces R247 which initially stacks to W179 and contacts the ZnF cysteine thiolates by forming two hydrogen bonds between its side chain and the S atom of C268 and one hydrogen bond between its NH main chain and the S atom of C245 in the ZnF native structure. Residues W179 and R247 are clearly involved in the second shell of the Zn-coordination sphere of *Ll*Fpg by establishing NH—S hydrogen bonds (from residue backbone and/or basic side chain with Cysteine thiolates) (Supplementary Table S3, Figure 7). Both experimental and theoretical studies show that such interactions tend to suppress and/or significantly decrease the inherent reactivity of Zn-bound thiolates in ‘structural’ ZnFs (especially those with 4 cysteines) by decreasing the apparent negative charge of the ZnF core (57–59). For example, one intramolecular NH—S hydrogen bond can decrease the rate of thiolate-alkylation more than 30 times (60,61). Thus, a comparison between the structures of native and oxidized ZnF suggests that the first effect associated with the binding of 2TX to W179 is probably the increase in the nucleophilicity of ZnF-thiolates by disrupting thiolate ligand interactions such as those mediated by R247. In both structures obtained in the presence of 2TX, R247 has been displaced (Supplementary Figure S3). If we assume that the reversible binding of 2TX is achieved in this way (Step 1, Figure 7), W179 appears to be a key residue for the binding of 2TX to *Ll*Fpg. This assumption is also supported by the observation that all ZnF-Fpg/Nei DNA glycosylases have an aromatic residue at the position of W179 in αE of their H2TH motifs (W, Y and F for *Ll*Fpg, *h*NEIL2 and *h*NEIL3, respectively) (Supplementary Figure S1d). Thus, the binding of 2TX to this conserved aromatic residue of the H2TH motif can be directly connected to the ability of the thiopurine to inhibit all ZnF-containing Fpg/Nei DNA glycosylases. In a second step (Step 2, Figure 7), the precise binding mode of 2TX to W179 leads to exquisitely expose the thio (thione/thiolate) group of 2TX to the the ZnF activated thiolates of C268 and C248. Because of their vicinity, both sulfur atoms are readily oxidized in the intermolecular disulfide bond observed in the crystal structures according to a thiolate/disulfide interchange mechanism. Such an oxidation of the free 2TX molecule in its dimeric disulfide form without Fpg is strongly disadvantaged due to the stability of its thione tautomer which predominates in aqueous solution. Through binding to Fpg, 2TX can also be activated by shifting from its stable thione to its active thiolate tautomer as has been observed for mercaptobenzimadazole absorption onto gold nanoparticles (62) (2TX-S*, Figure 7). At the same time, the thiolate oxidation destabilizes the Zn-coordination sphere and finally results in the loss of Zn ion and the formation of an intra-disulfide bond between the most buried C245 and C265 (E*I or E*SI, Figure 7).

At this stage of our knowledge, the precise redox chemistry of the ZnF oxidation by 2TX remains to be elucidated and does not exclude other possible oxidation products after the loss of zinc whose nature strongly depends on the redox potential of the medium. It is possible that, before its release, the Zn^2+^ ion could also play a role in this redox reaction. From this point of view, it is interesting to note that a strong reducer power (1 mM of tris(2-carboxyethyl)phosphine, TCEP, present in the stock Fpg solution) does not prevent the oxidation of Fpg ZnF by 2TX in the crystal structures nor in solution. A similar conclusion can be drawn when 2TX-induced zinc release is analyzed in the presence of an excess of various thio-reducers such as β-mercaptoethanol (β-SH), dithiothreitol (DTT) and reduced glutathion (GSH) (Supplementary Figure S6). As expected, reducers do not affect the structural integrity of Fpg ZnF followed by the PAR assay (lanes 3, 4, 7, 8, 11 and 12, Supplementary Figure S6a). This is not surprising since such reducers are often used in Fpg stock buffers and known to protect the enzyme from oxidation. Interestingly, the presence of thio-reducers in a 1/1 molar ratio with 2TX (10 mM/10 mM against 5 μM of *Ll*Fpg) partially protects the enzyme by decreasing the apparent Zn-release velocity (by about 1.5–2 times, Supplementary Figure S6b) without, however, abolishing 2TX-induced oxidation process. This experiment clearly confirmed the oxidative action of 2TX on the enzyme. Thus, 2TX is able to attack Zn-sulfur coordination bonds of ZnF-Fpg also in the presence of GSH, the major redox form of glutathione in normal cellular conditions. This result is consistent with the observation that 2TX can operate *in cellulo* by significantly inhibiting the 5-OHC-DNA glycosylase activity partly associated with *h*NEIL2 (Figure 6).

In order to test the molecular mechanism for inhibition proposed in Figure 7, the key residues of *Ll*Fpg suspected to play a role in 2TX-mediated inhibition in our 3D structures were targeted by site-directed mutagenesis. Four *Ll*Fpg variants were constructed: W179A, R247G, C248GH and C268H. The aim of constructing these point mutations was to (i) disrupt the 2TX binding site (W179A), (ii) increase the C268 thiolate reactivity (R247G) or (iii)inactivate the reactive sites C248 or C268 by replacing C to H (C248GH, which mimics the situation found in *h*NEIL2, Supplementary Figure S1c; C268H). Whereas all variants appear functional *in vivo* by complementing the mutator phenotype of the *E. coli fpg^−^* strain (Supplementary Figure S7a), only R247G and C248GH could be overproduced in *E. coli*, purified to homogeneity and characterized structurally and functionally. The crystal structures of R247G and C248GH bound to 14-mer THF-DNA have been solved and indicate that these point mutations do not affect the overall structure of ZnF as compared to the wild-type enzyme (Supplementary Figure S7b,c, Supplementary Tables S2 and S3). To evaluate the 2TX sensitivity of R247G and C248GH, we used the PAR assay (Table 1). 2TX is able to extract the zinc ion from ZnF of R247G 3-times faster than it does with the wild-type enzyme. Assuming that the PAR assay is another manner to estimate the inhibition efficiency of the enzyme (Figure 5), the variant R247G appears more sensitive than the wild-type enzyme. As expected by 3D structures, the suppression of NH—S bonds from the second coordination shell of ZnF increases the reactivity of ZnF thiolates in the variant R247G and probably also the accessibility to W179 for binding (Supplementary Table S2). In contrast, the variant C248GH appears about two times less sensitive to 2TX than the wild type (Table 1). As seen in the crystal structure of [WT/THF]-2TX (Figure 2b), this suggests that C248 is one of the reactive targets of 2TX but not the only one since C248GH remains sensitive to 2TX. This result is in perfect agreement with the crystal structure of [WT/Bz]-2TX (Figure 2c) showing that C268 is another reactive target for 2TX and with the observation that *h*NEIL2 containing CHC2 type of ZnF (Supplementary Figure S1c) is also inhibited by 2TX (Figure 5, Table 1). Biochemical and structural experiments combined with site-directed mutagenesis strongly support the proposed molecular mechanism by which Fpg and the other ZnF-containing Fpg/Nei DNA glycosylases are inhibited by 2TX (Figure 7). In this mechanism, the chemical reactivity of 2TX results from its peculiar interaction with its enzyme binding site (conserved aromatic site), whereas the free molecule in solution can be considered hardly reactive in its major oxothione tautomer (Figure 1a). This molecular mechanism is very different from the one proposed for the inhibition of Myelopexoxidase (MPO, an enzyme involved in oxidative stress during inflammation) by 2TX and its N3-alkylated-derivatives (63,64). In this case, the inhibition leads to the oxidation of the drug resulting in a covalent adduct between the sulfur atom of 2TX and the enzyme heme prosthestic group by following a complex radical chemistry used naturally by MPO to oxidize several of its substrates (here and differently from our case, 2TX-derivatives mimic true substrates). In this last case, it was also proposed that the thio-tautomer nature of the 2TX-derivatives determines their interaction with MPO and thus their reactivity against the enzyme.

## CONCLUSION

Because of the cancer therapeutic potential, the search for DNA glycosylase inhibitors is a very active field mainly focused on the discovery of small molecules expected to mimic the substrate by binding in the enzyme active site or at an allosteric site preventing the removing of the damage base (*competitive* inhibitors). In this work, we deciphered at the atomic level the structural and/or functional determinants required for the specific inhibition of ZnF Fpg/Nei DNA glycosylases by the *uncompetitive* inhibitor 2TX. 3D structures revealed that after its specific binding 2TX reacts with the enzyme by inducing the oxidation of ZnF which results in an irreversible inhibition associated with the loss of zinc ion. This study exemplifies that the special ZnF of Fpg/Nei DNA glycosylases can been seen as selective template for small ligand reactivity as it has been proposed for other ZnF proteins (65). Overall, the data presented herein highlight the structural and functional determinants for inhibition and establish a foundation for the rational design of new and more effective 2TX-derivatives taking into account the electrostatic and steric constraints highlighted by 3D structures. Contrary to *competitive* inhibitors characterized to date for DNA glycosylases (66–69), the interest of 2TX resides in its ability to target both the free enzymes and the enzymes bound to DNA (searching their substrates) and in the irreversible character of inhibition. Furthermore, the possibility of inhibiting a cell-cycle regulated DNA glycosylase such as *h*NEIL3 (a ZnF-Fpg/Nei DNA glycosylase, Supplementary Figure S1c) that is required in proliferating cells appears promising for future medicinal chemistry development in cancer therapy strategies. Since 2TX works on Fpg/Nei DNA glycosylases *in cellulo* without apparent toxicity, it will now be important to evaluate its selectivity (and that of its future derivatives) by studying its potential action on other Zn finger proteins and DNA glycosylases from other structural superfamilies.

## ACCESSION NUMBERS

The coordinates and structure factors have been deposited in the Protein Data Bank in Europe (PDBe) with id codes 4PDG, 4PDI, 4PCZ and 4PD2 (Supplementary Table S1).

## SUPPLEMENTARY DATA

Supplementary Data are available at NAR Online.

SUPPLEMENTARY DATA
