# Supplementary material for: Zinc finger oxidation of Fpg/Nei DNA glycosylases by 2-thioxanthine: biochemical and X-ray structural characterization
Source: Nucleic Acids Res. 2014 Aug 20;42(16):10748–61. doi: 10.1093/nar/gku613 (PMC4176347; doi:10.1093/nar/gku613)
Supplement: SUPPLEMENTARY DATA [file supp_42_16_10748__index.html]

Zinc finger oxidation of Fpg/Nei DNA glycosylases by 2-thioxanthine: biochemical and X-ray structural characterization — Zinc finger oxidation of Fpg/Nei DNA glycosylases by 2-thioxanthine: biochemical and X-ray structural characterization — SUPPLEMENTARY DATA 

# Zinc finger oxidation of Fpg/Nei DNA glycosylases by 2-thioxanthine: biochemical and X-ray structural characterization

## SUPPLEMENTARY DATA

**Files in this Data Supplement:**

- SUPPLEMENTARY DATA
