# Supplementary material for: Zinc finger oxidation of Fpg/Nei DNA glycosylases by 2-thioxanthine: biochemical and X-ray structural characterization
Source: Nucleic Acids Res. 2014 Aug 20;42(16):10748–61. doi: 10.1093/nar/gku613 (PMC4176347; doi:10.1093/nar/gku613)
Supplement: SUPPLEMENTARY DATA [file supp_gku613_nar-01194-m-2014-File010.pdf]

Supporting Information

Zinc finger oxidation of Fpg/Nei DNA glycosylases by 2-thioxanthine: biochemical and X-ray structural characterization

Artur BIELA, Franck COSTE, Françoise CULARD, Martine GUERIN, Stéphane GOFFINONT, Karola GASTEIGER, Jarosław CIEŚLA, Alicja WINCZURA, Zygmunt KAZIMIERCZUK, Didier GASPARUTTO, Thomas CARELL, Barbara TUDEK and Bertrand CASTAING

SI table captions

Table S1: X-ray data collection and refinement statistics

[a] $R_{\text{merge}} = \sum_h \sum_i |I_{h,i} - \langle I \rangle_h| / \sum_h \sum_i I_{h,i}$ where $\langle I \rangle_h$ is the mean intensity of the symmetry-equivalent reflections.

[b] $R_{\text{work}} = \sum_h ||F_o| - |F_c|| / \sum_h |F_o|$, where F_o and F_c are the observed and calculated structure factor amplitudes, respectively, for reflection h.

[c] R_{free} is the R value for a subset of 5% of the reflection data, which were not included in the crystallographic refinement.

Table S2 : Accessible surface areas of zinc finger cysteine thiolates

The sulfur accessible surface area (SASA in Å2) of cysteine thiolate (SG) was calculated using POPS (Fraternali et al., 2002) with a 1.8Å radius probe (van der Waals radius of sulfur atom) for wt and variants of *Ll*Fpg (with or without 2TX) and for other ZnF-Fpg/Nei DNA glycosylases. The PDB id code of each structure is indicated in parentheses. The sulfur atom used as probe is assumed to mimic the thione group of 2TX.

Table S3: Thiolate direct shielding by H-bonds (max 3.6 Å)

SI figure captions

Figure S1: Structures of the Fpg/Nei DNA glycosylases

(a) and **(b)** Overview of the structures of *Ll*Fpg and *Mv*Nei1 bound to DNA (pdb id codes 1PM5 and 3A42, respectively). The two DNA binding domains zinc/zincless (*Ll*Fpg/*Mv*Nei1) finger (ZnF/ZnLF) and H2TH motif are highlighted in *blue* and *magenta*, respectively. The catalytic P1 emerging at the interface between both globular domains of the enzymes is indicated. The strictly conserved arginine residue (R260 in *Ll*Fpg) of the b-hairpin loop is also shown. **(c)** and **(d)** Primary structure alignment and Superimposition of X-ray structures of DNA binding domains of Fpg/Nei DNA glycosylases (1PM5 and 2EA0 for *Ll*Fpg, and *Ec*Nei bound to DNA, respectively and 1TDH for free *h*NEIL1). The color code is *green* for *Ll*Fpg, *ligth blue* for *Ec*Nei and *purple* for *h*NEIL1. *Ll*, *Bst*, *Tt*, *Ec* and *h* are for *Lactococcus lactis*, *Bacillus stearothermophilus*, *Thermus thermophilus*, *Escherichia coli* and human, respectively. Cysteine-coordinating Zn^{2+} are highlighted by *white* letters on either *red* or *black* background. Strictly conserved arginine of the *β9-β10* loop is underlined in *white* letters on

grey background. The residue numbers indicated are those of *Ll*Fpg. *Grey* triangles indicate the point mutations tested in this work.

<u>Figure S2</u> : Oligonucleotides used in this study
(a) *Structures of substrate- and substrate analogue-containing DNA* 8-oxoG = 8-oxoguanine, Hyd = 5-hydroxy-5-methylhydanthoin, 5-OHC = 5-hydroxycytosine, THF = tetrahydrofuran and Bz-cFapyG = carbanucleoside analogue of 2,6-diamino-4-hydroxy-5-benzylformamidopyrimidine. **(b)** *Oligonucleotide sequences* DNA duplexes were obtained by hybridizing each modified strand (X, Y or Z) with its complementary strand “C”. 14-mer DNA duplexes were used in X-ray experiments, PAR assays and EMSAs, 24-mer and 34-mer DNA duplexes in enzyme activity. “X” is for THF or Bz-cFapyG in X-ray structure analysis and for THF in EMSA. “Y” is for 8-oxoG and Hyd in enzyme activity assays and “W” is for C or G as indicated. “Z” is for 5-OHC for glycosylase assay with cell free extracts.

<u>Figure S3</u>: Stereo views of a superimposition of the native and oxidized Fpg zinc finger
The same color code defined in Figure 2 was used: in *green*, the native ZnF observed in the crystal structure of *Ll*Fpg bound to 14-mer THF-DNA; in *blue* and *magenta*, oxidized forms of the zinc finger observed in the crystal structure of *Ll*Fpg bound to 14-mer DNA duplexes containing either THF or Bz-cFapyG nucleoside analogs after 2TX-treatment of the preformed complexes, respectively. See also the legend of Fig.2.

<u>Figure S4</u>: Effect of DMSO on the DNA binding activity of *Ll*Fpg
(a) *EMSA*. Binding experiments were carried out as described elsewhere (Castaing et al., 1999). 0.1 nM of radiolabelled THF-14-mer DNA duplex (Table S1, Fig. S3) was incubated 20 min at 4°C with 0, 0.25, 0.5, 1, 2, 4, 8, 16 nM of LlFpg with (+) or without (-) DMSO (8% final concentration). Resulting mixtures were then analyzed by EMSA. **(b)** *Apparent dissociation constants ($K_D$app)*. Indicated values correspond to the mean values obtained from three independent experiments.

<u>Figure S5</u>: Effect of different nucleobases on the Hyd-DNA glycosylase activity of Fpg/Nei DNA glycosylases
The excision of 5-hydroxy-5-methylhydanthoine (Hyd) contained in 24-mer single- or double-stranded oligonucleotides was analyzed in single-turnover condition in the presence of increased concentrations of G, X, 2TX, 8-oxoG and FapyG (nucleobase abreviations were defined in Fig.1). **(a)** Excision of Hyd opposite C by *Ll*Fpg, **(b)** and **(c)** Excision of Hyd opposite G by *Ec*Nei and *h*NEIL1, respectively and **(d)** Excision of Hyd in single-stranded 24-mer oligonucleotide by *h*NEIL2.

<u>Figure S6</u>: Effect of reducers on 2TX-induced release of zinc from *Ll*Fpg
(a) *Zn release in the presence of thioreducers* and **(b)** *Apparent kinetic constants (k_{obs})* in the presence of thioreducers. PAR assays were performed as described in the « Materials and Methods » section. DNA is for 14-mer THF-DNA, bSH for b-mercaptoethanol, DTT for dithiothreitol and GSH for reduced glutathione. Thioreducers were in a 1/1 molar ratio with 2TX and data plotted in (a) correspond to10 min incubation time at 20°C.

<u>Figure S7</u>: Structural and functional characterization of LlFpg zinc finger variants (a) *Frequencies of spontaneous mutation to rifampicin resistance in E. coli BH990 strain expressing different variants of LlFpg proteins* Complementation of the spontaneous mutator phenotype of BH990 (*fpg mutY*) transformed by different recombinant pFlag plamids expressing no enzyme (NO), the *L. lactis* wild type Fpg enzyme (WT) and the variants

W179A, R247G, C248GH and C268H of *Ll*Fpg were analyzed by determining the frequency of rifampicin-resistant cells in four independent experiments. Average values (+/-SD) of Rif revertants per 10^8 cells are reported as mutation frequency. ZnF structures of LlFpg variants R247G and C248GH. **(b)** and **(c)** *Zinc finger structures of R247G and C248GH, respectively* Native ZnF of wild type LlFpg (in green) is superimposed on that of R247G (in light brown, a) or C248GH (in grey, b). Side chains of R247 and W179 and of Zn2+ coordinating residues are indicated.

	[WT/THF]-2TX	[WT/Bz]-2TX	[R247G /THF]	[C248GH/THF]
**Data collection**				
Radiation source		SOLEIL PX-1		ESRF ID23-1
Wavelength (Å)	0.98011	1.54980	0.98011	0.98400
Spacegroup		$P\,4_12_12$		
Cell dimensions $a = b, c$ (Å)	90.55, 139.35	91.54, 140.97	91.72, 142.32	91.46, 140.39
Resolution range (Å)	43.06-2.40 (2.53-2.40)	47.67-2.10 (2.22-2.10)	47.93-1.70 (1.79-1.70)	47.56-1.65 (1.74-1.65)
Unique reflections	23,099 (3,334)	35,667 (5,089)	65,739 (8,486)	70,733 (10,318)
Completeness (%)	99.1 (99.5)	100.0 (99.8)	97.4 (88.2)	96.1 (96.4)
Redundancy	4.8 (4.9)	13.7 (11.1)	3.6 (3.3)	4.9 (4.8)
R_{merge} [a]	0.080 (0.871)	0.103 (0.705)	0.095 (0.411)	0.058 (0.705)
Mean $<I/\sigma(I)>$	16.2 (2.0)	17.0 (3.0)	8.4 (2.6)	14.2 (2.2)
Anomalous completeness (%)	-	99.9 (99.5)	-	-
Anomalous multiplicity	-	7.1 (5.7)	-	-
DelAnom correlation between half-sets	-	0.094 (0.006)	-	-
**Refinement**				
Resolution range (Å)	43.06-2.40	47.67-2.10	47.94-1.70	45.728-1.65
No. of reflections	23,079	35,595	65,702	70,703
R_{work} [b] / R_{free} [c]	0.163/0.193	0.160/0.190	0.157/0.182	0.154/0.179
No. of atoms				
Protein	2,101	2,138	2,192	2,174
DNA	557	576	568	557
2TX	11	11	-	0
Glycerol	6	0	12	0
Water	58	289	453	368
Average B values (Å^2)				
Protein	47.7	34.9	28.6	30.6
DNA	68.7	50.6	39.6	50.4
2TX	88.9	91.2	-	-
Glycerol	54.3	-	26.3	-
Water	46.5	44.6	40.4	42.7
Rmsd				
Bond lengths (Å)	0.012	0.008	0.019	0.017
Bond angles (°)	1.281	1.075	1.825	1.680
Ramachandran value (%)				
Favoured / Allowed / Outliers	97.7 / 2.3 / 0.0	97.4 / 2.6 / 0.0	96.4 / 3.6 / 0.0	98.2 / 1.8 / 0.0
Protein Data Bank ID code	4PDG	4PDI	4PCZ	4PD2

	***Ll*Fpg**					***Ec*Fpg**		***Tt*Fpg**		***Ec*Nei**		***Mm*NEIL3**	
	[WT/ THF] **(1PM5)**	***[WT/THF]-2TX*** *(this work)*	***[WT/Bz]-2TX*** *(this work)*	***[R247G/THF]*** *(this work)*	***[C248GH/THF]*** *(this work)*	**(1K82)**		**(1EE8)**		**(2EA0)**		**(3W0F)**	
**C245**(SG)	0.9	1.4	1.4	1.1	1.1	**C243**(SG)	1.2	**C238**(SG)	1.2	**C237**(SG)	0.8	**C255**(SG)	1.3
**C248**(SG)	3.1	21.4	59.4	2.7	-	**C246**(SG)	2.2	**C241**(SG)	3.1	**C240**(SG)	1.9	**C258**(SG)	1.8
**H249**(NE2)	-	-	-	-	1.9	-	-	-	-	-	-	-	-
**C265**(SG)	0.9	1.4	1.3	0.6	0.9	**C263**(SG)	1.0	**C258**(SG)	1.0	**C257**(SG)	0.8	**C277**(SG)	0.8
**C268**(SG)	2.3	5.2	1.9	2.9	1.6	**C266**(SG)	4.4	**C261**(SG)	3.6	**C260**(SG)	1.3	**C280**(SG)	3.3

Protein		Thiolate	donor	Distance (Å)
**LlFpg**				
	WT			
		S(C245)	N(R247)	3.36
			NE1(W179)	3.40
		S(C248)	N(A250)	3.23
		S(C265)	N(C268)	3.24
			N(V267)	3.52
		S(C268)	NE(R247)	3.45
			NH2(R247)	3.53
	R247G			
		S(C245)	N(G247)	3.21
			NE1(W179)	3.34
			N(C248)	3.56
		S(C248)	N(A250)	3.42
		S(C265)	N(C268)	3.35
			N(V267)	3.38
		S(C268)	O(H_2O172)	3.13
			O(H_2O170)	3.21
	C248GH			
		S(C245)	N(R247)	3.47
			NE1(W179)	3.38
		S(C266)	N(V268)	3.41
			N(C269)	3.44
		S(C269)	NH2(R247)	3.33
**EcFpg**				
		S(C243)	N(V245)	3.36
			N(C246)	3.46
			O(HOH561)	3.5
		S(C246)	OG1(T248)	3.24
			N(T248)	3.39
		S(C263)	N(C266)	3.36
		S(C266)	-	-
**EcNei**				
		S(C237)	N(R239)	3.33
			NE1(W176)	3.51
		S(C240)	N(S242)	3.53
		S(C257)	N(G259)	3.31
		S(C260)	NH2(R239)	3.22
			NE(R239)	3.52
**MmNEIL3**				
		S(C255)	N(Q257)	3.18
		S(C258)	OG(S260)	2.96
			N(S260)	3.56
		S(C277)	N(C280)	3.34
			N(H279)	3.45
		S(C280)	OE1(Q257)	3.20

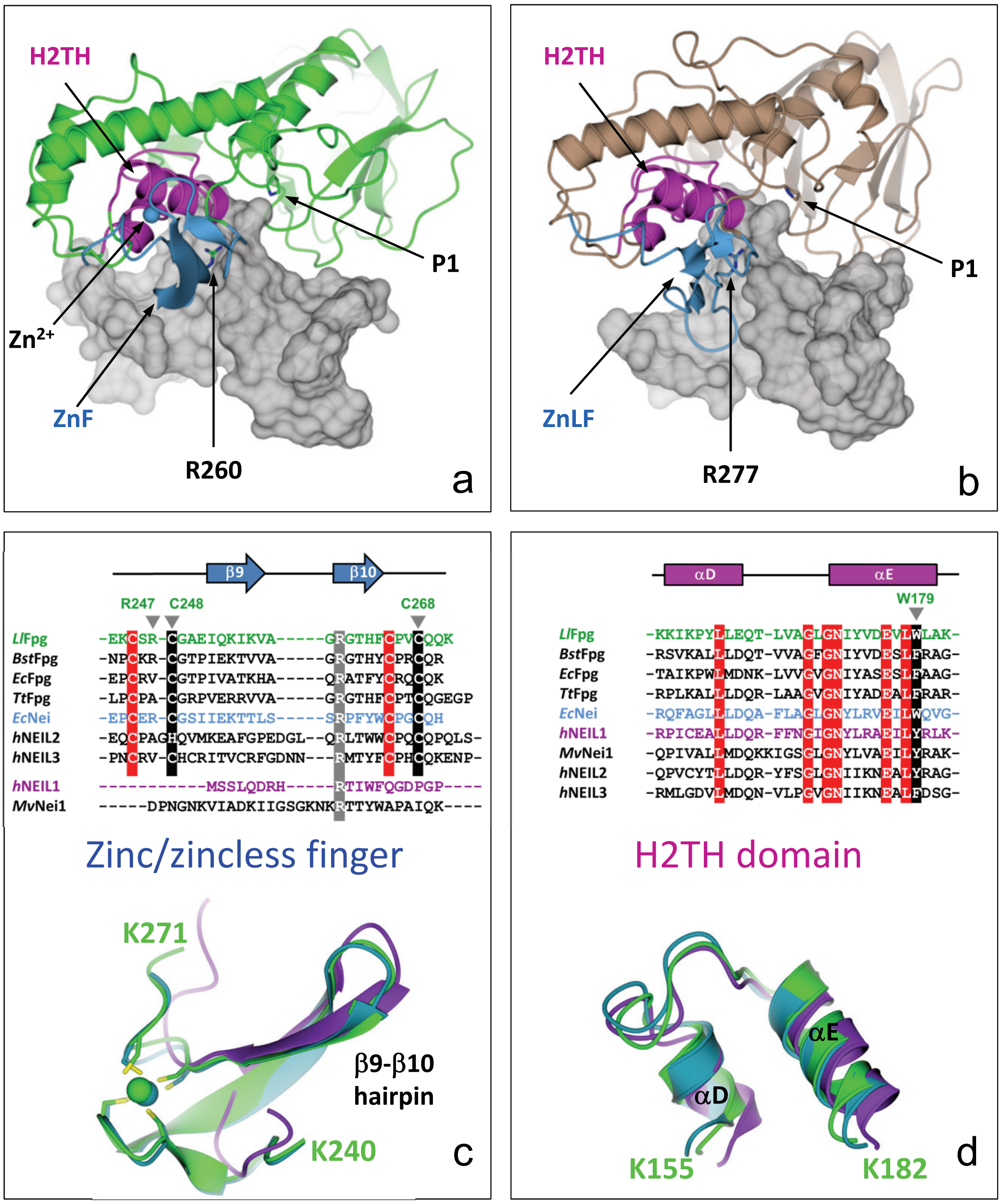

Biela *et al. (2014) Figure Sup* **Figure S1**

a

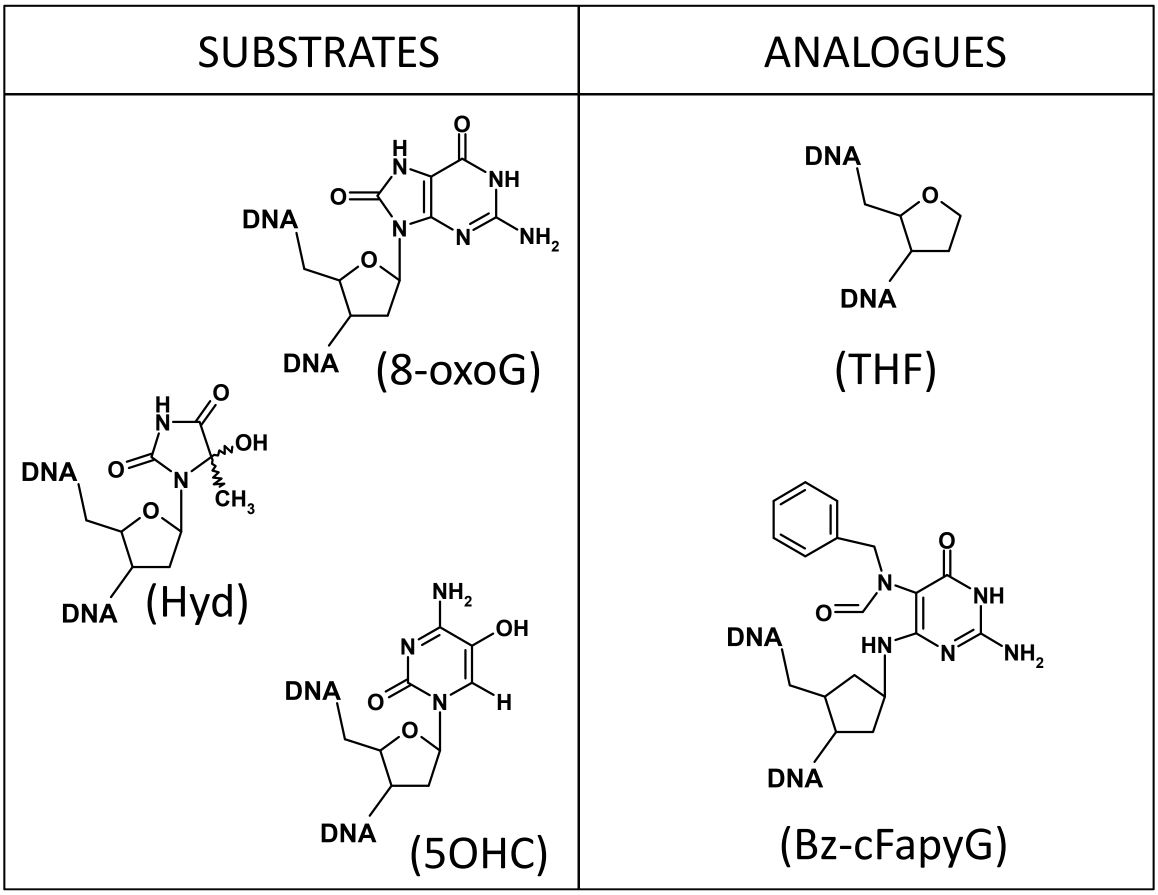

b

name	Sequence (5'–>3')	DNA duplexes
14-mer X	CTCTTT**X**TTTCTCG	14-mer X-DNA
14-mer C	GCGAGAAACAAAGA	
24-mer Y	GGCGCCTCTTT**Y**TTTCTCGCCGGG	24-mer Y-DNA
24-mer C	CCCGGCGAGAAA**W**AAAGAGGCGCC	
34-mer Z	CTGCAGCTGATGCGC**Z**GTACGGATCCCCGGGTAC	34-mer Z-DNA
34-mer C	GTACCCGGGGATCCGTACCGCGCATCAGCTGCAG	

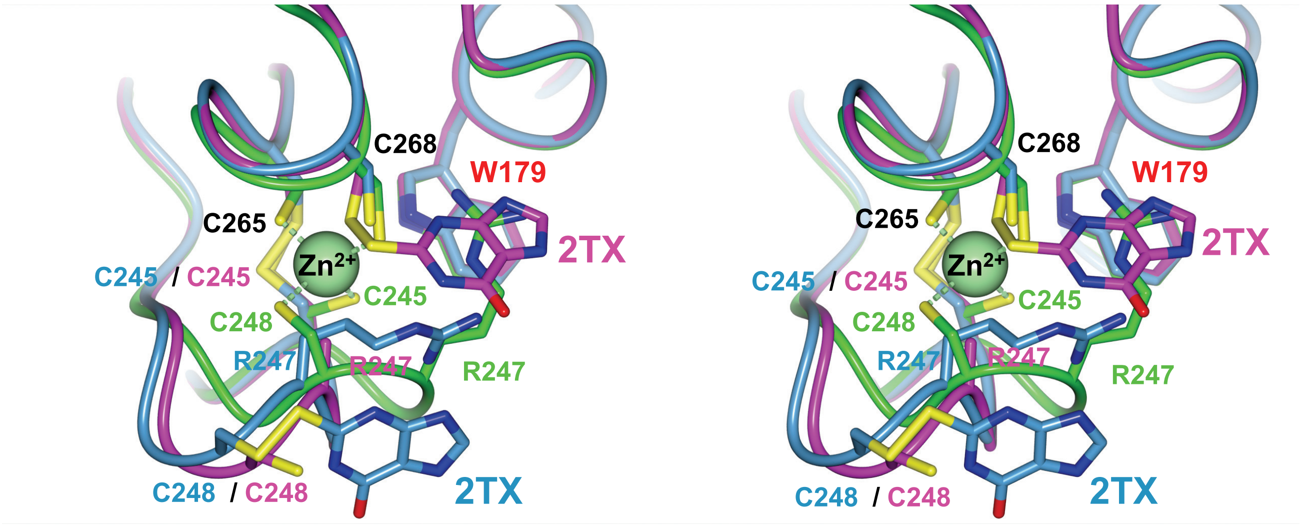

Biela *et al. (2014) Figure Sup** **Figure S3**

a

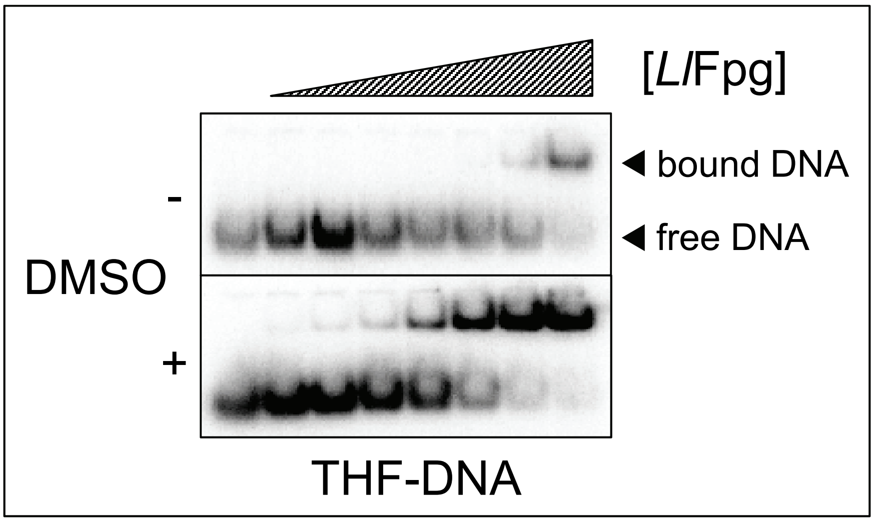

b

DNA duplexes	$K_D$app (nM)	
	(-) DMSO	*(+) DMSO*
THF-DNA	10 ±1	3 ±0.6

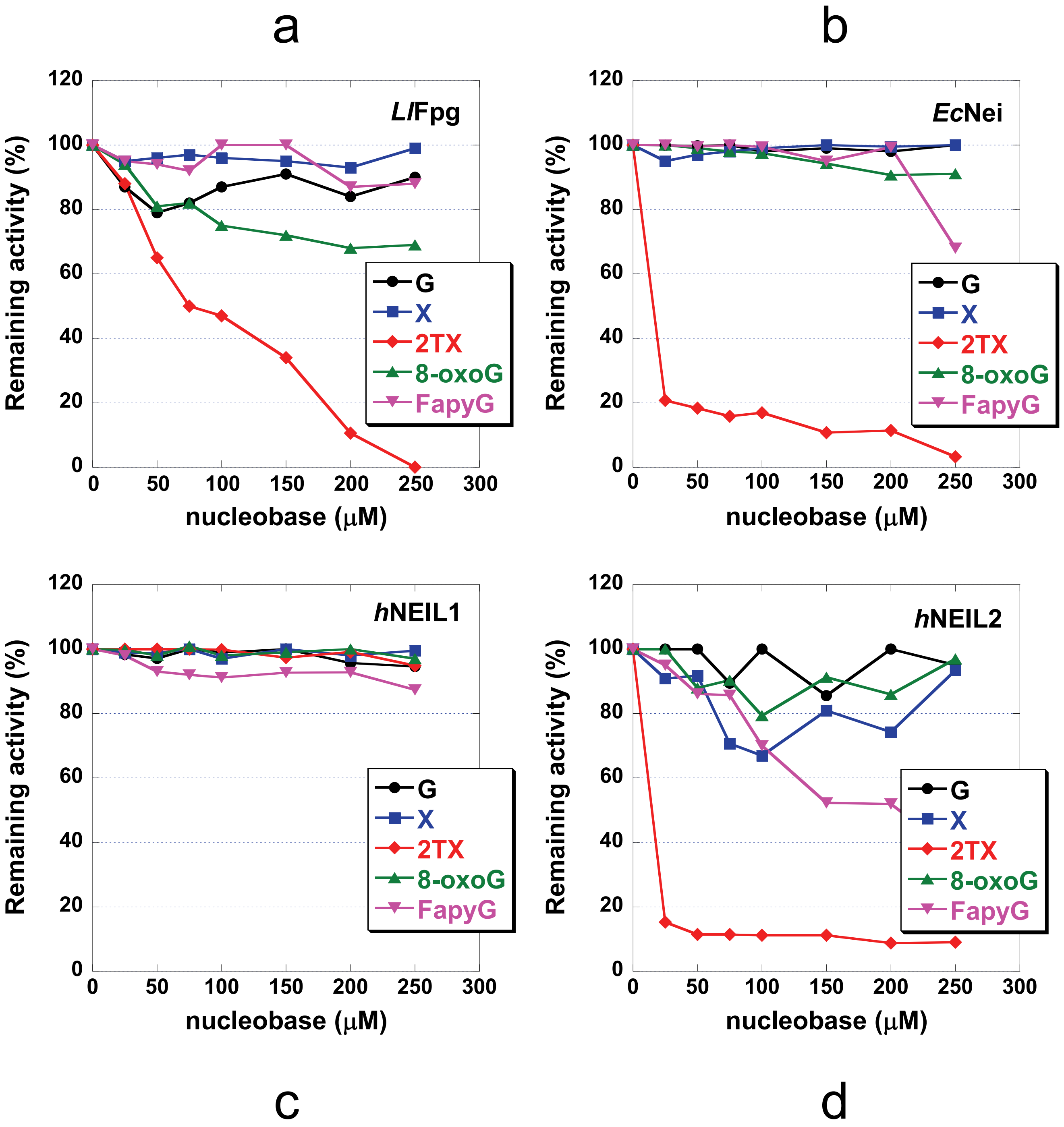

Biela *et al. (2014) Figure Sup* **Figure S5**

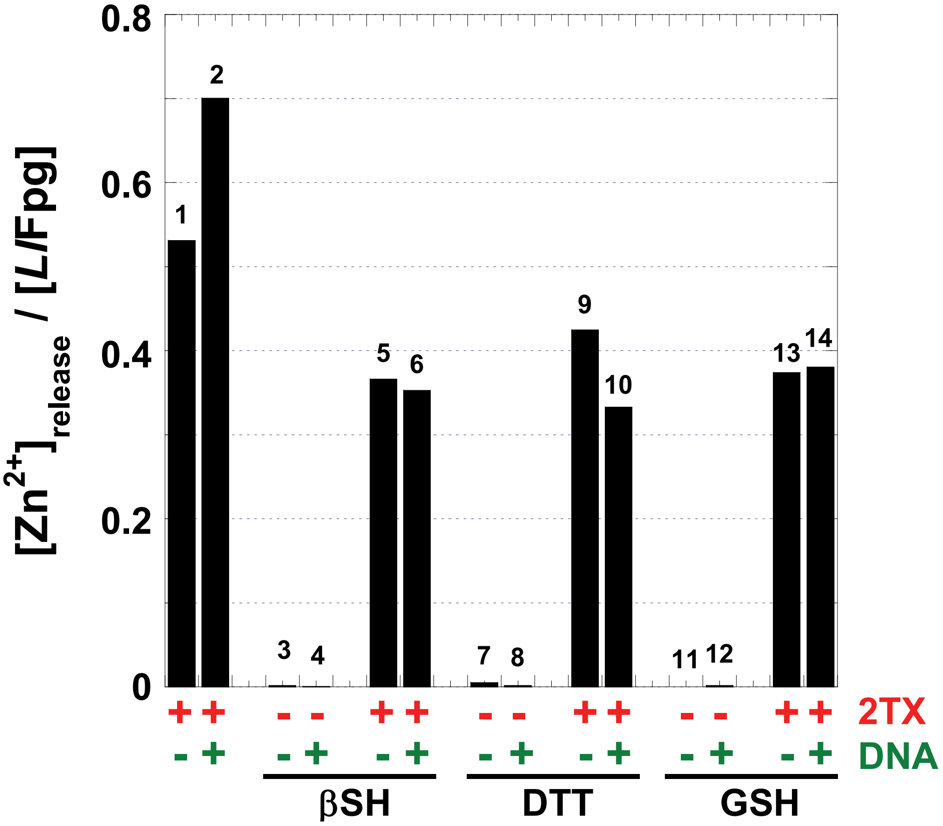

a

	k_{obs} ± SD (min^{-1})	
	(-) DNA	*(+) DNA*
2TX	0.073 ± 0.001	0.113 ± 0.004
2TX + βSH	0. 040 ± 0.002	0. 040 ± 0.002
2TX + DTT	0. 042 ± 0.002	0. 031 ± 0.002
2TX + GSH	0. 035 ± 0.002	0. 037 ± 0.002

b

Biela *et al. (2014) Figure Sup* **Figure S6**

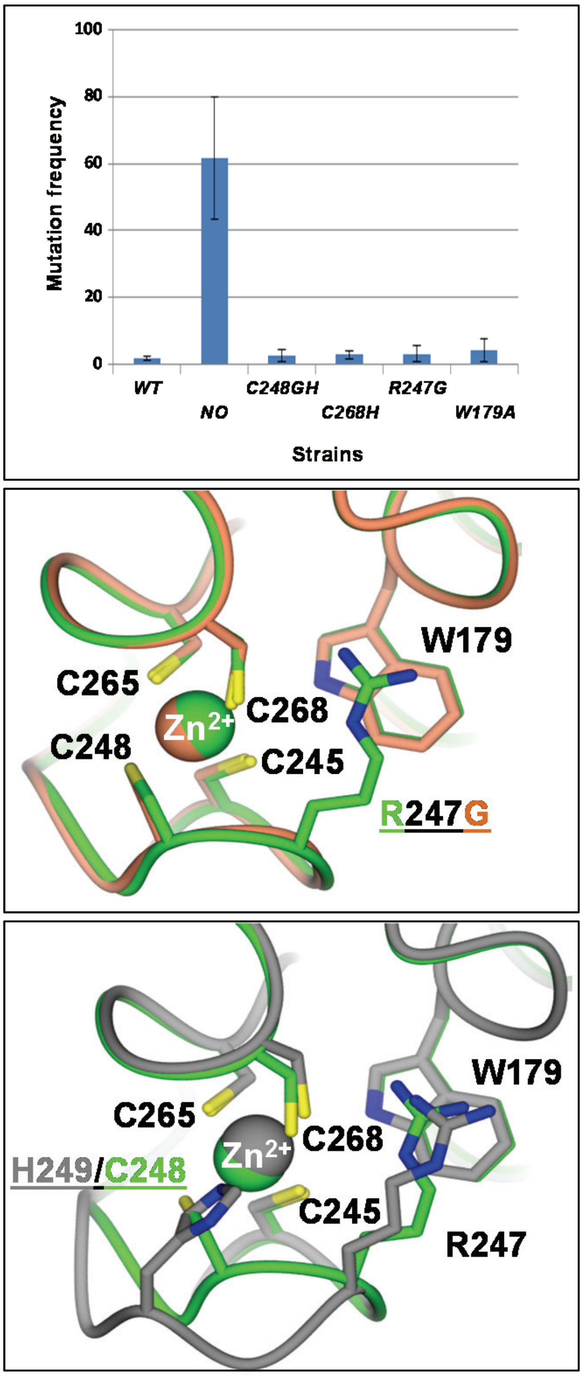

a

b

c

Biela *et al. (2014) Figure Sup**Figure S7**

Full wwPDB X-ray Structure Validation Report (i)

Apr 22, 2014 – 06:42 PM EDT

PDB ID	:	4PCZ
Title	:	Crystal structure of a complex between R247G LlFPG mutant and a THF containing DNA
Authors	:	Coste, F.; Castaing, B.
Deposited on	:	2014-04-17
Resolution	:	1.70 Å(reported)

DISCLAIMER
This is a preliminary version of the new style of wwPDB validation report.
We welcome your comments at validation@mail.wwpdb.org
A user guide is available at http://wwpdb.org/ValidationPDFNotes.html

CONFIDENTIAL VALIDATION REPORT

The following versions of software and data (see references) were used in the production of this report:

MolProbity	:	4.02b-467
Mogul	:	1.16 November 2013
Xtriage (Phenix)	:	dev-1439
EDS	:	stable22683
Percentile statistics	:	21963
Refmac	:	5.8.0049
CCP4	:	6.3.0 (Settle)
Ideal geometry (proteins)	:	Engh & Huber (2001)
Ideal geometry (DNA, RNA)	:	Parkinson et. al. (1996)
Validation Pipeline (wwPDB-VP)	:	stable22683

1 Overall quality at a glance (i)

The reported resolution of this entry is 1.70 Å.

Percentile scores (ranging between 0-100) for global validation metrics of the entry are shown in the following graphic. The table shows the number of entries on which the scores are based.

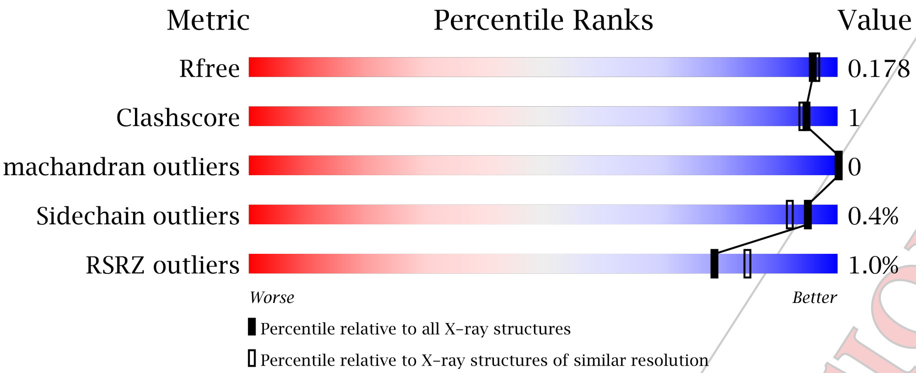

Metric	Whole archive (#Entries)	Similar resolution (#Entries, resolution range(Å))
R_{free}	66092	2456 (1.70-1.70)
Clashscore	79885	2929 (1.70-1.70)
Ramachandran outliers	78287	2878 (1.70-1.70)
Sidechain outliers	78261	2878 (1.70-1.70)
RSRZ outliers	66119	2456 (1.70-1.70)

The table below summarises the geometric issues observed across the polymeric chains and their fit to the electron density. The red, orange, yellow and green segments on the lower bar indicate the fraction of residues that contain outliers for >=3, 2, 1 and 0 types of geometric quality criteria. The upper red bar (where present) indicates the fraction of residues that have poor fit to the electron density.

Mol	Chain	Length	Quality of chain
1	A	271	
2	B	14	
3	C	14	

CONFIDENTIAL VALIDATION REPORT

2 Entry composition (i)

There are 6 unique types of molecules in this entry. The entry contains 3225 atoms, of which 0 are hydrogen and 0 are deuterium.

In the tables below, the ZeroOcc column contains the number of atoms modelled with zero occupancy, the AltConf column contains the number of residues with at least one atom in alternate conformation and the Trace column contains the number of residues modelled with at most 2 atoms.

- Molecule 1 is a protein called Formamidopyrimidine-DNAglycosylase.

Mol	Chain	Residues	Atoms					ZeroOcc	AltConf	Trace
			Total	C	N	O	S			
1	A	271	2191	1401	374	408	8	0	5	0

There are 2 discrepancies between the modelled and reference sequences:

Chain	Residue	Modelled	Actual	Comment	Reference
A	?	-	ASP	DELETION	UNP P42371
A	247	GLY	ARG	engineered mutation	UNP P42371

- Molecule 2 is a DNA chain called DNA (5'-D(*CP*TP*CP*TP*TP*TP*(3DR)P*TP*TP*TP*CP*TP*CP*G)-3').

Mol	Chain	Residues	Atoms					ZeroOcc	AltConf	Trace
			Total	C	N	O	P			
2	B	14	277	136	33	94	14	0	1	0

- Molecule 3 is a DNA chain called DNA (5'-D(*GP*CP*GP*AP*GP*AP*AP*AP*CP*AP*AP*AP*GP*A)-3').

Mol	Chain	Residues	Atoms					ZeroOcc	AltConf	Trace
			Total	C	N	O	P			
3	C	14	291	138	66	74	13	0	0	0

- Molecule 4 is ZINC ION (three-letter code: ZN) (formula: Zn).

Mol	Chain	Residues	Atoms		ZeroOcc	AltConf
			Total	Zn		
4	A	1	1	1	0	0

- Molecule 5 is GLYCEROL (three-letter code: GOL) (formula: $C_3H_8O_3$).

CONFIDENTIAL VALIDATION REPORT

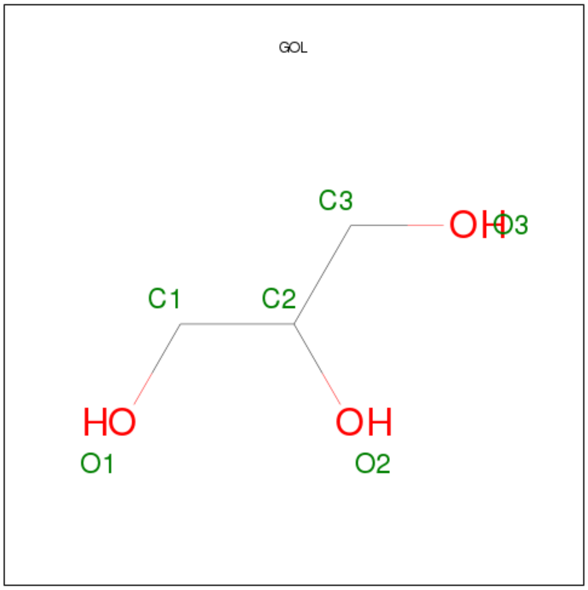

Mol	Chain	Residues	Atoms	ZeroOcc	AltConf
5	A	1	Total C O<br>12 6 6	0	1

- Molecule 6 is water.

Mol	Chain	Residues	Atoms	ZeroOcc	AltConf
6	A	375	Total O<br>375 375	0	0
6	B	43	Total O<br>43 43	0	0
6	C	35	Total O<br>35 35	0	0

CONFIDENTIAL VALIDATION REPORT

3 Residue-property plots (i)

These plots are drawn for all protein, RNA and DNA chains in the entry. The first graphic for a chain summarises the proportions of errors displayed in the second graphic. The second graphic shows the sequence view annotated by issues in geometry and electron density. Residues are color-coded according to the number of geometric quality criteria for which they contain at least one outlier: green = 0, yellow = 1, orange = 2 and red = 3 or more. A red dot above a residue indicates a poor fit to the electron density (RSRZ > 2). Stretches of 2 or more consecutive residues without any outlier are shown as a green connector. Residues present in the sample, but not in the model, are shown in grey.

- Molecule 1: Formamidopyrimidine-DNAglycosylase

Chain A: 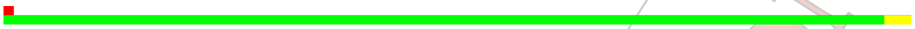

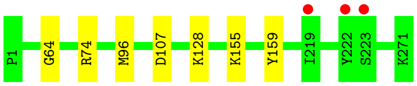

- Molecule 2: DNA (5’-D(*CP*TP*CP*TP*TP*TP*(3DR)P*TP*TP*TP*CP*TP*CP*G)-3’)

Chain B:

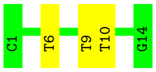

- Molecule 3: DNA (5’-D(*GP*CP*GP*AP*GP*AP*AP*AP*CP*AP*AP*AP*GP*A)-3’)

Chain C:

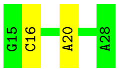

CONFIDENTIAL VALIDATION REPORT

4 Data and refinement statistics

Property	Value	Source
Space group	P 41 21 2	Depositor
Cell constants<br>a, b, c, α, β, γ	91.72Å 91.72Å 142.32Å<br>90.00° 90.00° 90.00°	Depositor
Resolution (Å)	47.94 – 1.70<br>47.93 – 1.70	Depositor<br>EDS
% Data completeness<br>(in resolution range)	94.3 (47.94-1.70)<br>97.1 (47.93-1.70)	Depositor<br>EDS
R_{merge}	0.10	Depositor
R_{sym}	(Not available)	Depositor
$< I/\sigma(I) >$ [1]	2.08 (at 1.70Å)	Xtriage
Refinement program	PHENIX (phenix.refine: 1.8.4_1496)	Depositor
R, R_{free}	0.157 , 0.181<br>0.155 , 0.178	Depositor<br>DCC
R_{free} test set	3350 reflections (5.10%)	DCC
Wilson B-factor (Å^2)	24.3	Xtriage
Anisotropy	0.182	Xtriage
Bulk solvent k_{sol}(e/Å^3), B_{sol}(Å^2)	0.37 , 45.7	EDS
Estimated twinning fraction	No twinning to report.	Xtriage
L-test for twinning	$< \|L\| > = 0.49$, $< L^2 > = 0.32$	Xtriage
Outliers	0 of 65708 reflections	Xtriage
F_o,F_c correlation	0.97	EDS
Total number of atoms	3225	wwPDB-VP
Average B, all atoms (Å^2)	32.0	wwPDB-VP

Xtriage's analysis on translational NCS is as follows: *The largest off-origin peak in the Patterson function is 3.96% of the height of the origin peak. No significant pseudotranslation is detected.*

[1]Intensities estimated from amplitudes.

CONFIDENTIAL VALIDATION REPORT

5 Model quality (i)

5.1 Standard geometry (i)

Bond lengths and bond angles in the following residue types are not validated in this section: GOL, ZN, 3DR

The Z score for a bond length (or angle) is the number of standard deviations the observed value is removed from the expected value. A bond length (or angle) with $|Z| > 5$ is considered an outlier worth inspection. RMSZ is the root-mean-square of all Z scores of the bond lengths (or angles).

Mol	Chain	Bond lengths		Bond angles	
		RMSZ	#|Z| >5	RMSZ	#|Z| >5
1	A	0.89	0/2232	0.93	3/2998 (0.1%)
2	B	1.33	1/301 (0.3%)	1.47	2/459 (0.4%)
3	C	1.45	1/330 (0.3%)	1.22	1/508 (0.2%)
All	All	1.02	2/2863 (0.1%)	1.04	6/3965 (0.2%)

All (2) bond length outliers are listed below:

Mol	Chain	Res	Type	Atoms	Z	Observed(Å)	Ideal(Å)
3	C	20	DA	C3'-O3'	-6.00	1.36	1.44
2	B	9	DT	C5-C7	5.41	1.53	1.50

All (6) bond angle outliers are listed below:

Mol	Chain	Res	Type	Atoms	Z	Observed(^{o})	Ideal(^{o})
2	B	10	DT	OP2-P-O3'	6.27	118.99	105.20
1	A	74	ARG	NE-CZ-NH1	6.24	123.42	120.30
1	A	96	MET	CA-CB-CG	6.17	123.79	113.30
1	A	107	ASP	CB-CG-OD1	5.65	123.39	118.30
3	C	16	DC	OP2-P-O3'	5.30	116.86	105.20
2	B	6	DT	C5-C4-O4	-5.17	121.28	124.90

There are no chirality outliers.

There are no planarity outliers.

5.2 Close contacts (i)

In the following table, the Non-H and H(model) columns list the number of non-hydrogen atoms and hydrogen atoms in the chain respectively. The H(added) column lists the number of hydrogens added by MolProbity. The Clashes column lists the number of clashes within the asymmetric unit,

CONFIDENTIAL VALIDATION REPORT

and the number in parentheses is this value normalized per 1000 atoms of the molecule in the chain. The Symm-Clashes column gives symmetry related clashes, in the same way as for the Clashes column.

Mol	Chain	Non-H	H(model)	H(added)	Clashes	Symm-Clashes
1	A	2191	0	0	3	0
2	B	277	0	0	0	0
3	C	291	0	0	0	0
4	A	1	0	0	0	0
5	A	12	0	16	0	0
6	A	375	0	0	3	0
6	B	43	0	0	0	0
6	C	35	0	0	0	0
All	All	3225	0	16	3	0

Clashscore is defined as the number of clashes calculated for the entry per 1000 atoms (including hydrogens) of the entry. The overall clashscore for this entry is 1.

All (3) close contacts within the same asymmetric unit are listed below.

Atom-1	Atom-2	Distance(Å)	Clash(Å)
1:A:128:LYS:NZ	6:A:402:HOH:O	2.38	0.56
1:A:155:LYS:NZ	6:A:744:HOH:O	2.47	0.47
1:A:64:GLY:O	6:A:774:HOH:O	2.20	0.46

There are no symmetry-related clashes.

5.3 Torsion angles

5.3.1 Protein backbone (i)

In the following table, the Percentiles column shows the percent Ramachandran outliers of the chain as a percentile score with respect to all X-ray entries followed by that with respect to entries of similar resolution. The Analysed column shows the number of residues for which the backbone conformation was analysed, and the total number of residues.

Mol	Chain	Analysed	Favoured	Allowed	Outliers	Percentiles	
1	A	274/271 (101%)	263 (96%)	11 (4%)	0	100	100

There are no Ramachandran outliers to report.

CONFIDENTIAL VALIDATION REPORT

5.3.2 Protein sidechains (i)

In the following table, the Percentiles column shows the percent sidechain outliers of the chain as a percentile score with respect to all X-ray entries followed by that with respect to entries of similar resolution. The Analysed column shows the number of residues for which the sidechain conformation was analysed, and the total number of residues.

Mol	Chain	Analysed	Rotameric	Outliers	Percentiles	
1	A	237/238 (100%)	236 (100%)	1 (0%)	95	92

All (1) residues with a non-rotameric sidechain are listed below:

Mol	Chain	Res	Type
1	A	159	TYR

Some sidechains can be flipped to improve hydrogen bonding and reduce clashes. There are no such sidechains identified.

5.3.3 RNA (i)

There are no RNA chains in this entry.

5.4 Non-standard residues in protein, DNA, RNA chains (i)

1 non-standard protein/DNA/RNA residue is modelled in this entry.

In the following table, the Counts columns list the number of bonds (or angles) for which Mogul statistics could be retrieved, the number of bonds (or angles) that are observed in the model and the number of bonds (or angles) that are defined in the chemical component dictionary. The Link column lists molecule types, if any, to which the group is linked. The Z score for a bond length (or angle) is the number of standard deviations the observed value is removed from the expected value. A bond length (or angle) with $|Z| > 2$ is considered an outlier worth inspection. RMSZ is the root-mean-square of all Z scores of the bond lengths (or angles).

Mol	Type	Chain	Res	Link	Bond lengths			Bond angles		
					Counts	RMSZ	$\#\|Z\| > 2$	Counts	RMSZ	$\#\|Z\| > 2$
2	3DR	B	7	2	9,11,12	3.90	4 (44%)	11,14,17	1.71	2 (18%)

In the following table, the Chirals column lists the number of chiral outliers, the number of chiral centers analysed, the number of these observed in the model and the number defined in the chemical component dictionary. Similar counts are reported in the Torsion and Rings columns. '-' means no outliers of that kind were identified.

CONFIDENTIAL VALIDATION REPORT

Mol	Type	Chain	Res	Link	Chirals	Torsions	Rings
2	3DR	B	7	2	-	0/4/15/16	0/1/1/1

All (4) bond length outliers are listed below:

Mol	Chain	Res	Type	Atoms	Z	Observed(Å)	Ideal(Å)
2	B	7	3DR	O5'-C5'	-8.92	1.31	1.44
2	B	7	3DR	C2'-C3'	-5.58	1.42	1.52
2	B	7	3DR	P-OP1	4.31	1.51	1.46
2	B	7	3DR	C3'-C4'	-2.27	1.46	1.53

All (2) bond angle outliers are listed below:

Mol	Chain	Res	Type	Atoms	Z	Observed(^{o})	Ideal(^{o})
2	B	7	3DR	O4'-C4'-C5'	4.38	119.29	109.58
2	B	7	3DR	O4'-C1'-C2'	-2.09	102.13	106.59

There are no chirality outliers.

There are no torsion outliers.

There are no ring outliers.

5.5 Carbohydrates (i)

There are no carbohydrates in this entry.

5.6 Ligand geometry (i)

Of 3 ligands modelled in this entry, 1 is monoatomic - leaving 2 for Mogul analysis.

In the following table, the Counts columns list the number of bonds (or angles) for which Mogul statistics could be retrieved, the number of bonds (or angles) that are observed in the model and the number of bonds (or angles) that are defined in the chemical component dictionary. The Link column lists molecule types, if any, to which the group is linked. The Z score for a bond length (or angle) is the number of standard deviations the observed value is removed from the expected value. A bond length (or angle) with $|Z| > 2$ is considered an outlier worth inspection. RMSZ is the root-mean-square of all Z scores of the bond lengths (or angles).

Mol	Type	Chain	Res	Link	Bond lengths			Bond angles		
					Counts	RMSZ	$\#\|Z\| > 2$	Counts	RMSZ	$\#\|Z\| > 2$
5	GOL	A	302[A]	-	5,5,5	0.75	0	5,5,5	0.78	0
5	GOL	A	302[B]	-	5,5,5	0.57	0	5,5,5	0.75	0

CONFIDENTIAL VALIDATION REPORT

In the following table, the Chirals column lists the number of chiral outliers, the number of chiral centers analysed, the number of these observed in the model and the number defined in the chemical component dictionary. Similar counts are reported in the Torsion and Rings columns. '-' means no outliers of that kind were identified.

Mol	Type	Chain	Res	Link	Chirals	Torsions	Rings
5	GOL	A	302[A]	-	-	0/4/4/4	0/0/0/0
5	GOL	A	302[B]	-	-	0/4/4/4	0/0/0/0

There are no bond length outliers.

There are no bond angle outliers.

There are no chirality outliers.

There are no torsion outliers.

There are no ring outliers.

5.7 Other polymers (i)

There are no such residues in this entry.

5.8 Polymer linkage issues

There are no chain breaks in this entry.

CONFIDENTIAL VALIDATION REPORT

6 Fit of model and data ⓘ

6.1 Protein, DNA and RNA chains ⓘ

In the following table, the column labelled '#RSRZ> 2' contains the number (and percentage) of RSRZ outliers, followed by percent RSRZ outliers for the chain as percentile scores relative to all X-ray entries and entries of similar resolution. The OWAB column contains the minimum, median, 95th percentile and maximum values of the occupancy-weighted average B-factor per residue. The column labelled 'Q< 0.9' lists the number of (and percentage) of residues with an average occupancy less than 0.9.

Mol	Chain	Analysed	<RSRZ>	#RSRZ>2			OWAB(Å^2)	Q<0.9
1	A	271/271 (100%)	-0.51	3 (1%)	77	83	17, 25, 47, 115	1 (0%)
2	B	14/14 (100%)	-0.31	0	100	100	21, 34, 46, 46	0
3	C	14/14 (100%)	-0.47	0	100	100	25, 40, 64, 71	0
All	All	299/299 (100%)	-0.49	3 (1%)	79	85	17, 26, 51, 115	1 (0%)

All (3) RSRZ outliers are listed below:

Mol	Chain	Res	Type	RSRZ
1	A	219	ILE	3.0
1	A	223	SER	2.6
1	A	222	TYR	2.1

6.2 Non-standard residues in protein, DNA, RNA chains ⓘ

In the following table, the Atoms column lists the number of modelled atoms in the group and the number defined in the chemical component dictionary. LLDF column lists the quality of electron density of the group with respect to its neighbouring residues in protein, DNA or RNA chains. The B-factors column lists the minimum, median, 95th percentile and maximum values of B factors of atoms in the group. The column labelled 'Q< 0.9' lists the number of atoms with occupancy less than 0.9.

Mol	Type	Chain	Res	Atoms	RSR	LLDF	B-factors(Å^2)	Q<0.9
2	3DR	B	7	11/12	0.07	0.95	18,25,27,31	0

6.3 Carbohydrates ⓘ

There are no carbohydrates in this entry.

CONFIDENTIAL VALIDATION REPORT

6.4 Ligands (i)

In the following table, the Atoms column lists the number of modelled atoms in the group and the number defined in the chemical component dictionary. LLDF column lists the quality of electron density of the group with respect to its neighbouring residues in protein, DNA or RNA chains. The B-factors column lists the minimum, median, 95th percentile and maximum values of B factors of atoms in the group. The column labelled 'Q< 0.9' lists the number of atoms with occupancy less than 0.9.

Mol	Type	Chain	Res	Atoms	RSR	LLDF	B-factors($Å^2$)	Q<0.9
5	GOL	A	302[B]	6/6	0.10	1.04	17,21,22,22	6
5	GOL	A	302[A]	6/6	0.10	0.95	28,30,33,34	6
4	ZN	A	301	1/1	0.05	-0.96	27,27,27,27	0

6.5 Other polymers (i)

There are no such residues in this entry.

CONFIDENTIAL VALIDATION REPORT

Full wwPDB X-ray Structure Validation Report (i)

Apr 22, 2014 – 07:51 PM EDT

PDB ID	:	4PD2
Title	:	Crystal structure of a complex between a C248GH LlFpg mutant and a THF containing DNA
Authors	:	Coste, F.; Castaing, B.
Deposited on	:	2014-04-17
Resolution	:	1.65 Å(reported)

DISCLAIMER
This is a preliminary version of the new style of wwPDB validation report.
We welcome your comments at validation@mail.wwpdb.org
A user guide is available at http://wwpdb.org/ValidationPDFNotes.html

CONFIDENTIAL VALIDATION REPORT

The following versions of software and data (see references) were used in the production of this report:

MolProbity	:	4.02b-467
Mogul	:	1.16 November 2013
Xtriage (Phenix)	:	dev-1439
EDS	:	stable22683
Percentile statistics	:	21963
Refmac	:	5.8.0049
CCP4	:	6.3.0 (Settle)
Ideal geometry (proteins)	:	Engh & Huber (2001)
Ideal geometry (DNA, RNA)	:	Parkinson et. al. (1996)
Validation Pipeline (wwPDB-VP)	:	stable22683

1 Overall quality at a glance ⓘ

The reported resolution of this entry is 1.65 Å.

Percentile scores (ranging between 0-100) for global validation metrics of the entry are shown in the following graphic. The table shows the number of entries on which the scores are based.

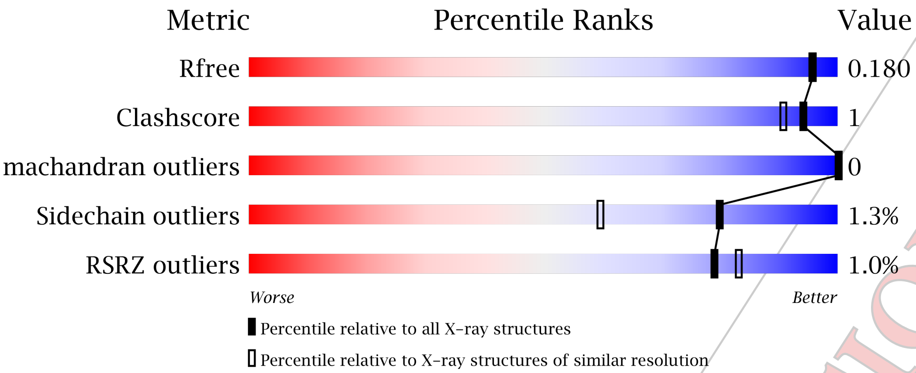

Metric	Whole archive (#Entries)	Similar resolution (#Entries, resolution range(Å))
R_{free}	66092	1404 (1.68-1.64)
Clashscore	79885	1001 (1.66-1.66)
Ramachandran outliers	78287	1581 (1.68-1.64)
Sidechain outliers	78261	1580 (1.68-1.64)
RSRZ outliers	66119	1404 (1.68-1.64)

The table below summarises the geometric issues observed across the polymeric chains and their fit to the electron density. The red, orange, yellow and green segments on the lower bar indicate the fraction of residues that contain outliers for >=3, 2, 1 and 0 types of geometric quality criteria. The upper red bar (where present) indicates the fraction of residues that have poor fit to the electron density.

Mol	Chain	Length	Quality of chain
1	A	272	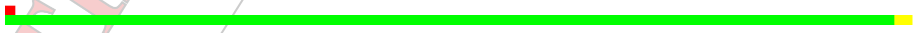
2	B	14	
3	C	14	

CONFIDENTIAL VALIDATION REPORT

2 Entry composition (i)

There are 5 unique types of molecules in this entry. The entry contains 3099 atoms, of which 0 are hydrogen and 0 are deuterium.

In the tables below, the ZeroOcc column contains the number of atoms modelled with zero occupancy, the AltConf column contains the number of residues with at least one atom in alternate conformation and the Trace column contains the number of residues modelled with at most 2 atoms.

- Molecule 1 is a protein called Formamidopyrimidine-DNAglycosylase.

Mol	Chain	Residues	Atoms					ZeroOcc	AltConf	Trace
			Total	C	N	O	S			
1	A	272	2173	1396	373	397	7	0	4	0

There are 3 discrepancies between the modelled and reference sequences:

Chain	Residue	Modelled	Actual	Comment	Reference
A	?	-	ASP	DELETION	UNP P42371
A	248	GLY	-	insertion	UNP P42371
A	249	HIS	CYS	ENGINEERED MUTATION	UNP P42371

- Molecule 2 is a DNA chain called DNA (5'-D(*CP*TP*CP*TP*TP*TP*(3DR)P*TP*TP*TP*CP*TP*CP*G)-3').

Mol	Chain	Residues	Atoms					ZeroOcc	AltConf	Trace
			Total	C	N	O	P			
2	B	14	266	131	33	89	13	0	0	0

- Molecule 3 is a DNA chain called DNA (5'-D(*GP*CP*GP*AP*GP*AP*AP*AP*CP*AP*AP*AP*GP*A)-3').

Mol	Chain	Residues	Atoms					ZeroOcc	AltConf	Trace
			Total	C	N	O	P			
3	C	14	291	138	66	74	13	0	0	0

- Molecule 4 is ZINC ION (three-letter code: ZN) (formula: Zn).

Mol	Chain	Residues	Atoms		ZeroOcc	AltConf
			Total	Zn		
4	A	1	1	1	0	0

- Molecule 5 is water.

CONFIDENTIAL VALIDATION REPORT

Mol	Chain	Residues	Atoms	ZeroOcc	AltConf
5	A	311	Total O<br>311 311	0	0
5	B	26	Total O<br>26 26	0	0
5	C	31	Total O<br>31 31	0	0

CONFIDENTIAL VALIDATION REPORT

3 Residue-property plots (i)

These plots are drawn for all protein, RNA and DNA chains in the entry. The first graphic for a chain summarises the proportions of errors displayed in the second graphic. The second graphic shows the sequence view annotated by issues in geometry and electron density. Residues are color-coded according to the number of geometric quality criteria for which they contain at least one outlier: green = 0, yellow = 1, orange = 2 and red = 3 or more. A red dot above a residue indicates a poor fit to the electron density (RSRZ > 2). Stretches of 2 or more consecutive residues without any outlier are shown as a green connector. Residues present in the sample, but not in the model, are shown in grey.

- Molecule 1: Formamidopyrimidine-DNAglycosylase

Chain A: 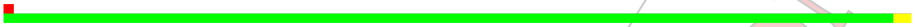

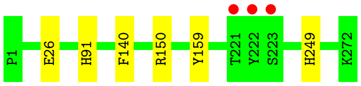

- Molecule 2: DNA (5'-D(*CP*TP*CP*TP*TP*TP*(3DR)P*TP*TP*TP*CP*TP*CP*G)-3')

Chain B:

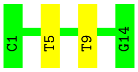

- Molecule 3: DNA (5'-D(*GP*CP*GP*AP*GP*AP*AP*AP*CP*AP*AP*AP*GP*A)-3')

Chain C:

There are no outlier residues recorded for this chain.

CONFIDENTIAL VALIDATION REPORT

4 Data and refinement statistics ⓘ

Property	Value	Source
Space group	P 41 21 2	Depositor
Cell constants<br>a, b, c, α, β, γ	91.46Å 91.46Å 140.39Å<br>90.00° 90.00° 90.00°	Depositor
Resolution (Å)	45.73 – 1.65<br>47.56 – 1.65	Depositor<br>EDS
% Data completeness<br>(in resolution range)	98.5 (45.73-1.65)<br>98.0 (47.56-1.65)	Depositor<br>EDS
R_{merge}	0.06	Depositor
R_{sym}	(Not available)	Depositor
$< I/\sigma(I) >$ [1]	1.81 (at 1.65Å)	Xtriage
Refinement program	PHENIX (phenix.refine: 1.8.4_1496)	Depositor
R, R_{free}	0.152 , 0.179<br>0.154 , 0.180	Depositor<br>DCC
R_{free} test set	3584 reflections (5.07%)	DCC
Wilson B-factor (Å^2)	24.4	Xtriage
Anisotropy	0.094	Xtriage
Bulk solvent k_{sol}(e/Å^3), B_{sol}(Å^2)	0.38 , 50.3	EDS
Estimated twinning fraction	No twinning to report.	Xtriage
L-test for twinning	$< \|L\| > = 0.48$, $< L^2 > = 0.32$	Xtriage
Outliers	0 of 70708 reflections	Xtriage
F_o,F_c correlation	0.97	EDS
Total number of atoms	3099	wwPDB-VP
Average B, all atoms (Å^2)	35.0	wwPDB-VP

Xtriage's analysis on translational NCS is as follows: *The largest off-origin peak in the Patterson function is 3.96% of the height of the origin peak. No significant pseudotranslation is detected.*

[1] Intensities estimated from amplitudes.

CONFIDENTIAL VALIDATION REPORT

5 Model quality (i)

5.1 Standard geometry (i)

Bond lengths and bond angles in the following residue types are not validated in this section: ZN, 3DR

The Z score for a bond length (or angle) is the number of standard deviations the observed value is removed from the expected value. A bond length (or angle) with $|Z| > 5$ is considered an outlier worth inspection. RMSZ is the root-mean-square of all Z scores of the bond lengths (or angles).

Mol	Chain	Bond lengths		Bond angles	
		RMSZ	$\#\|Z\| > 5$	RMSZ	$\#\|Z\| > 5$
1	A	0.79	0/2225	0.86	1/2994 (0.0%)
2	B	1.28	1/280 (0.4%)	1.39	1/427 (0.2%)
3	C	1.21	0/330	0.98	0/508
All	All	0.91	1/2835 (0.0%)	0.95	2/3929 (0.1%)

All (1) bond length outliers are listed below:

Mol	Chain	Res	Type	Atoms	Z	Observed(Å)	Ideal(Å)
2	B	9	DT	P-OP1	5.57	1.58	1.49

All (2) bond angle outliers are listed below:

Mol	Chain	Res	Type	Atoms	Z	Observed(^{o})	Ideal(^{o})
2	B	5	DT	O5'-P-OP2	-5.87	100.41	105.70
1	A	140	PHE	CB-CG-CD2	-5.34	117.06	120.80

There are no chirality outliers.

There are no planarity outliers.

5.2 Close contacts (i)

In the following table, the Non-H and H(model) columns list the number of non-hydrogen atoms and hydrogen atoms in the chain respectively. The H(added) column lists the number of hydrogens added by MolProbity. The Clashes column lists the number of clashes within the asymmetric unit, and the number in parentheses is this value normalized per 1000 atoms of the molecule in the chain. The Symm-Clashes column gives symmetry related clashes, in the same way as for the Clashes column.

CONFIDENTIAL VALIDATION REPORT

Mol	Chain	Non-H	H(model)	H(added)	Clashes	Symm-Clashes
1	A	2173	0	0	2	0
2	B	266	0	0	0	0
3	C	291	0	0	0	0
4	A	1	0	0	0	0
5	A	311	0	0	2	1
5	B	26	0	0	0	0
5	C	31	0	0	0	0
All	All	3099	0	0	2	1

Clashscore is defined as the number of clashes calculated for the entry per 1000 atoms (including hydrogens) of the entry. The overall clashscore for this entry is 1.

All (2) close contacts within the same asymmetric unit are listed below.

Atom-1	Atom-2	Distance(Å)	Clash(Å)
1:A:26:GLU:OE2	5:A:709:HOH:O	2.14	0.65
1:A:91:HIS:N	5:A:583:HOH:O	2.53	0.40

All (1) symmetry-related close contacts are listed below. The label for Atom-2 includes the symmetry operator and encoded unit-cell translations to be applied.

Atom-1	Atom-2	Distance(Å)	Clash(Å)
5:A:461:HOH:O	5:A:685:HOH:O[7_555]	2.16	0.04

5.3 Torsion angles

5.3.1 Protein backbone (i)

In the following table, the Percentiles column shows the percent Ramachandran outliers of the chain as a percentile score with respect to all X-ray entries followed by that with respect to entries of similar resolution. The Analysed column shows the number of residues for which the backbone conformation was analysed, and the total number of residues.

Mol	Chain	Analysed	Favoured	Allowed	Outliers	Percentiles	
1	A	274/272 (101%)	269 (98%)	5 (2%)	0	100	100

There are no Ramachandran outliers to report.

5.3.2 Protein sidechains (i)

In the following table, the Percentiles column shows the percent sidechain outliers of the chain as a percentile score with respect to all X-ray entries followed by that with respect to entries of

CONFIDENTIAL VALIDATION REPORT

similar resolution. The Analysed column shows the number of residues for which the sidechain conformation was analysed, and the total number of residues.

Mol	Chain	Analysed	Rotameric	Outliers	Percentiles	
1	A	229/239 (96%)	226 (99%)	3 (1%)	80	60

All (3) residues with a non-rotameric sidechain are listed below:

Mol	Chain	Res	Type
1	A	150	ARG
1	A	159	TYR
1	A	249	HIS

Some sidechains can be flipped to improve hydrogen bonding and reduce clashes. There are no such sidechains identified.

5.3.3 RNA ⓘ

There are no RNA chains in this entry.

5.4 Non-standard residues in protein, DNA, RNA chains ⓘ

1 non-standard protein/DNA/RNA residue is modelled in this entry.

In the following table, the Counts columns list the number of bonds (or angles) for which Mogul statistics could be retrieved, the number of bonds (or angles) that are observed in the model and the number of bonds (or angles) that are defined in the chemical component dictionary. The Link column lists molecule types, if any, to which the group is linked. The Z score for a bond length (or angle) is the number of standard deviations the observed value is removed from the expected value. A bond length (or angle) with $|Z| > 2$ is considered an outlier worth inspection. RMSZ is the root-mean-square of all Z scores of the bond lengths (or angles).

Mol	Type	Chain	Res	Link	Bond lengths			Bond angles		
					Counts	RMSZ	$\#\|Z\| > 2$	Counts	RMSZ	$\#\|Z\| > 2$
2	3DR	B	7	2	9,11,12	3.89	2 (22%)	11,14,17	1.66	3 (27%)

In the following table, the Chirals column lists the number of chiral outliers, the number of chiral centers analysed, the number of these observed in the model and the number defined in the chemical component dictionary. Similar counts are reported in the Torsion and Rings columns. '-' means no outliers of that kind were identified.

Mol	Type	Chain	Res	Link	Chirals	Torsions	Rings
2	3DR	B	7	2	-	0/4/15/16	0/1/1/1

CONFIDENTIAL VALIDATION REPORT

All (2) bond length outliers are listed below:

Mol	Chain	Res	Type	Atoms	Z	Observed(Å)	Ideal(Å)
2	B	7	3DR	O5'-C5'	-9.11	1.31	1.44
2	B	7	3DR	C2'-C3'	-6.70	1.40	1.52

All (3) bond angle outliers are listed below:

Mol	Chain	Res	Type	Atoms	Z	Observed(^{o})	Ideal(^{o})
2	B	7	3DR	O4'-C4'-C5'	2.91	116.03	109.58
2	B	7	3DR	C5'-C4'-C3'	-2.14	101.50	114.71
2	B	7	3DR	O4'-C4'-C3'	2.13	107.15	103.91

There are no chirality outliers.

There are no torsion outliers.

There are no ring outliers.

5.5 Carbohydrates (i)

There are no carbohydrates in this entry.

5.6 Ligand geometry (i)

Of 1 ligands modelled in this entry, 1 is monoatomic - leaving 0 for Mogul analysis.

There are no bond length outliers.

There are no bond angle outliers.

There are no chirality outliers.

There are no torsion outliers.

There are no ring outliers.

5.7 Other polymers (i)

There are no such residues in this entry.

5.8 Polymer linkage issues

There are no chain breaks in this entry.

CONFIDENTIAL VALIDATION REPORT

6 Fit of model and data (i)

6.1 Protein, DNA and RNA chains (i)

In the following table, the column labelled '#RSRZ> 2' contains the number (and percentage) of RSRZ outliers, followed by percent RSRZ outliers for the chain as percentile scores relative to all X-ray entries and entries of similar resolution. The OWAB column contains the minimum, median, 95th percentile and maximum values of the occupancy-weighted average B-factor per residue. The column labelled 'Q< 0.9' lists the number of (and percentage) of residues with an average occupancy less than 0.9.

Mol	Chain	Analysed	<RSRZ>	#RSRZ>2			OWAB(Å^2)	Q<0.9
1	A	272/272 (100%)	-0.58	3 (1%)	77	81	19, 27, 54, 130	0
2	B	14/14 (100%)	-0.04	0	100	100	24, 46, 68, 68	0
3	C	14/14 (100%)	-0.10	0	100	100	27, 57, 73, 74	0
All	All	300/300 (100%)	-0.54	3 (1%)	79	83	19, 28, 65, 130	0

All (3) RSRZ outliers are listed below:

Mol	Chain	Res	Type	RSRZ
1	A	221	THR	3.3
1	A	222	TYR	2.7
1	A	223	SER	2.3

6.2 Non-standard residues in protein, DNA, RNA chains (i)

In the following table, the Atoms column lists the number of modelled atoms in the group and the number defined in the chemical component dictionary. LLDF column lists the quality of electron density of the group with respect to its neighbouring residues in protein, DNA or RNA chains. The B-factors column lists the minimum, median, 95th percentile and maximum values of B factors of atoms in the group. The column labelled 'Q< 0.9' lists the number of atoms with occupancy less than 0.9.

Mol	Type	Chain	Res	Atoms	RSR	LLDF	B-factors(Å^2)	Q<0.9
2	3DR	B	7	11/12	0.07	1.04	20,26,32,35	0

6.3 Carbohydrates (i)

There are no carbohydrates in this entry.

CONFIDENTIAL VALIDATION REPORT

6.4 Ligands (i)

In the following table, the Atoms column lists the number of modelled atoms in the group and the number defined in the chemical component dictionary. LLDF column lists the quality of electron density of the group with respect to its neighbouring residues in protein, DNA or RNA chains. The B-factors column lists the minimum, median, 95th percentile and maximum values of B factors of atoms in the group. The column labelled 'Q< 0.9' lists the number of atoms with occupancy less than 0.9.

Mol	Type	Chain	Res	Atoms	RSR	LLDF	B-factors(Å^2)	Q<0.9
4	ZN	A	300	1/1	0.06	0.55	24,24,24,24	0

6.5 Other polymers (i)

There are no such residues in this entry.

CONFIDENTIAL VALIDATION REPORT

Full wwPDB X-ray Structure Validation Report (i)

Apr 28, 2014 – 05:28 PM EDT

PDB ID	:	4PDG
Title	:	Crystal structure of a complex between an inhibited LlFpg and a THF containing DNA
Authors	:	Coste, F.; Castaing, B.
Deposited on	:	2014-04-18
Resolution	:	2.40 Å(reported)

This is a full wwPDB validation report for a publicly released PDB entry.
We welcome your comments at validation@mail.wwpdb.org
A user guide is available at http://wwpdb.org/ValidationPDFNotes.html

The following versions of software and data (see references) were used in the production of this report:

MolProbity	:	4.02b-467
Mogul	:	1.16 November 2013
Xtriage (Phenix)	:	dev-1439
EDS	:	stable23106
Percentile statistics	:	21963
Refmac	:	5.8.0049
CCP4	:	6.3.0 (Settle)
Ideal geometry (proteins)	:	Engh & Huber (2001)
Ideal geometry (DNA, RNA)	:	Parkinson et. al. (1996)
Validation Pipeline (wwPDB-VP)	:	stable23106

1 Overall quality at a glance (i)

The reported resolution of this entry is 2.40 Å.

Percentile scores (ranging between 0-100) for global validation metrics of the entry are shown in the following graphic. The table shows the number of entries on which the scores are based.

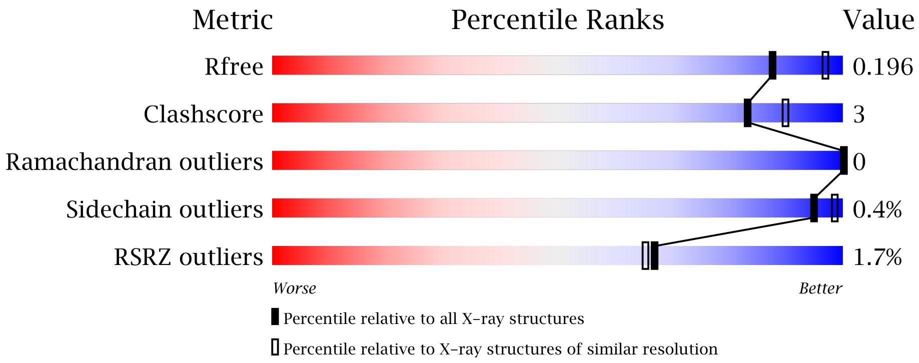

Metric	Whole archive (#Entries)	Similar resolution (#Entries, resolution range(Å))
R_{free}	66092	2207 (2.40-2.40)
Clashscore	79885	2789 (2.40-2.40)
Ramachandran outliers	78287	2736 (2.40-2.40)
Sidechain outliers	78261	2737 (2.40-2.40)
RSRZ outliers	66119	2210 (2.40-2.40)

The table below summarises the geometric issues observed across the polymeric chains and their fit to the electron density. The red, orange, yellow and green segments on the lower bar indicate the fraction of residues that contain outliers for >=3, 2, 1 and 0 types of geometric quality criteria. The upper red bar (where present) indicates the fraction of residues that have poor fit to the electron density.

Mol	Chain	Length	Quality of chain
1	A	271	
2	B	14	
3	C	14	

2 Entry composition (i)

There are 6 unique types of molecules in this entry. The entry contains 2733 atoms, of which 0 are hydrogen and 0 are deuterium.

In the tables below, the ZeroOcc column contains the number of atoms modelled with zero occupancy, the AltConf column contains the number of residues with at least one atom in alternate conformation and the Trace column contains the number of residues modelled with at most 2 atoms.

- Molecule 1 is a protein called Formamidopyrimidine-DNAglycosylase.

Mol	Chain	Residues	Atoms					ZeroOcc	AltConf	Trace
			Total	C	N	O	S			
1	A	266	2101	1347	360	386	8	0	0	0

- Molecule 2 is a DNA chain called DNA (5'-D(*CP*TP*CP*TP*TP*TP*(3DR)P*TP*TP*TP*CP*TP*CP*G)-3').

Mol	Chain	Residues	Atoms					ZeroOcc	AltConf	Trace
			Total	C	N	O	P			
2	B	14	266	131	33	89	13	0	0	0

- Molecule 3 is a DNA chain called DNA (5'-D(*GP*CP*GP*AP*GP*AP*AP*AP*CP*AP*AP*AP*GP*A)-3').

Mol	Chain	Residues	Atoms					ZeroOcc	AltConf	Trace
			Total	C	N	O	P			
3	C	14	291	138	66	74	13	0	0	0

- Molecule 4 is 2-sulfanyl-1,9-dihydro-6H-purin-6-one (three-letter code: 2ON) (formula: $C_5H_4N_4OS$).

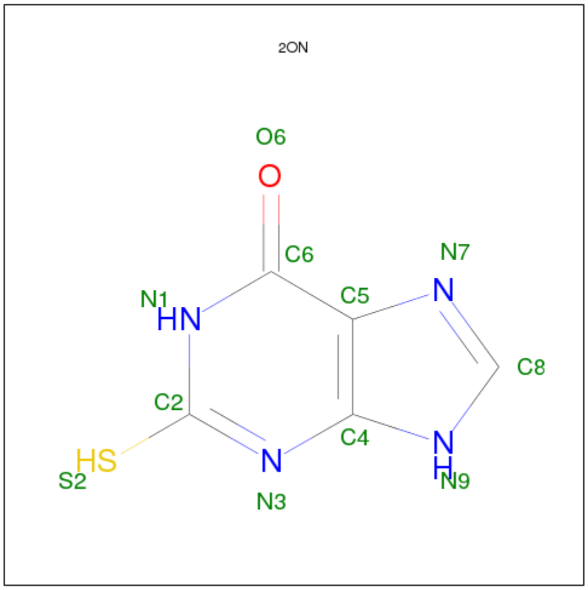

Mol	Chain	Residues	Atoms	ZeroOcc	AltConf
4	A	1	Total C N O S<br>11 5 4 1 1	0	0

- Molecule 5 is GLYCEROL (three-letter code: GOL) (formula: $C_3H_8O_3$).

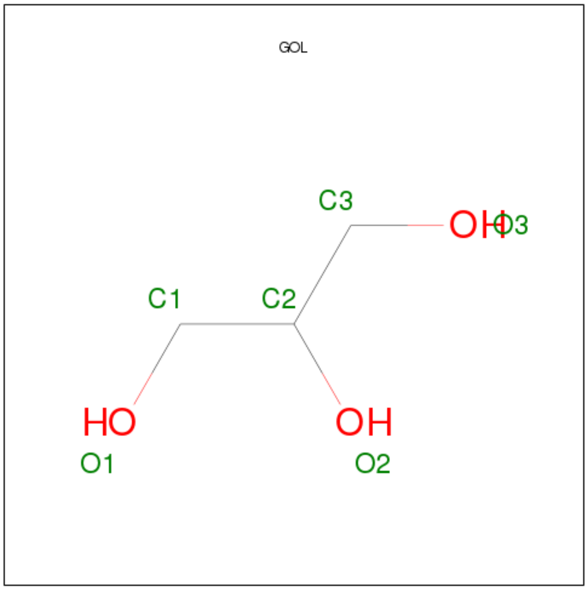

Mol	Chain	Residues	Atoms	ZeroOcc	AltConf
5	A	1	Total C O<br>6 3 3	0	0

- Molecule 6 is water.

Mol	Chain	Residues	Atoms	ZeroOcc	AltConf
6	A	50	Total O<br>50 50	0	0
6	B	5	Total O<br>5 5	0	0
6	C	3	Total O<br>3 3	0	0

3 Residue-property plots (i)

These plots are drawn for all protein, RNA and DNA chains in the entry. The first graphic for a chain summarises the proportions of errors displayed in the second graphic. The second graphic shows the sequence view annotated by issues in geometry and electron density. Residues are color-coded according to the number of geometric quality criteria for which they contain at least one outlier: green = 0, yellow = 1, orange = 2 and red = 3 or more. A red dot above a residue indicates a poor fit to the electron density (RSRZ > 2). Stretches of 2 or more consecutive residues without any outlier are shown as a green connector. Residues present in the sample, but not in the model, are shown in grey.

- Molecule 1: Formamidopyrimidine-DNAglycosylase

Chain A:

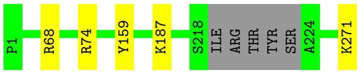

- Molecule 2: DNA (5'-D(*CP*TP*CP*TP*TP*TP*(3DR)P*TP*TP*TP*CP*TP*CP*G)-3')

Chain B: 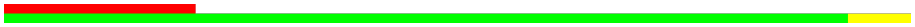

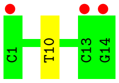

- Molecule 3: DNA (5'-D(*GP*CP*GP*AP*GP*AP*AP*AP*CP*AP*AP*AP*GP*A)-3')

Chain C: 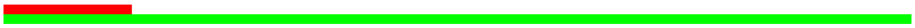

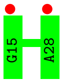

4 Data and refinement statistics (i)

Property	Value	Source
Space group	P 41 21 2	Depositor
Cell constants<br>a, b, c, α, β, γ	90.55Å 90.55Å 139.35Å<br>90.00° 90.00° 90.00°	Depositor
Resolution (Å)	43.06 – 2.40<br>43.06 – 2.40	Depositor<br>EDS
% Data completeness<br>(in resolution range)	99.1 (43.06-2.40)<br>98.6 (43.06-2.40)	Depositor<br>EDS
R_{merge}	(Not available)	Depositor
R_{sym}	(Not available)	Depositor
$< I/\sigma(I) >$ [1]	1.79 (at 2.39Å)	Xtriage
Refinement program	PHENIX (phenix.refine: 1.8.4_1496)	Depositor
R, R_{free}	0.163 , 0.193<br>0.164 , 0.196	Depositor<br>DCC
R_{free} test set	1183 reflections (5.13%)	DCC
Wilson B-factor (Å^2)	45.0	Xtriage
Anisotropy	0.227	Xtriage
Bulk solvent k_{sol}(e/Å^3), B_{sol}(Å^2)	0.43 , 52.6	EDS
Estimated twinning fraction	No twinning to report.	Xtriage
L-test for twinning	$< \|L\| > = 0.49$, $< L^2 > = 0.32$	Xtriage
Outliers	0 of 23087 reflections	Xtriage
F_o,F_c correlation	0.95	EDS
Total number of atoms	2733	wwPDB-VP
Average B, all atoms (Å^2)	52.0	wwPDB-VP

Xtriage's analysis on translational NCS is as follows: *The largest off-origin peak in the Patterson function is 4.10% of the height of the origin peak. No significant pseudotranslation is detected.*

[1] Intensities estimated from amplitudes.

5 Model quality (i)

5.1 Standard geometry (i)

Bond lengths and bond angles in the following residue types are not validated in this section: GOL, 3DR, 2ON

The Z score for a bond length (or angle) is the number of standard deviations the observed value is removed from the expected value. A bond length (or angle) with $|Z| > 5$ is considered an outlier worth inspection. RMSZ is the root-mean-square of all Z scores of the bond lengths (or angles).

Mol	Chain	Bond lengths		Bond angles	
		RMSZ	$\#\|Z\| >5$	RMSZ	$\#\|Z\| >5$
1	A	0.57	0/2138	0.64	0/2875
2	B	1.08	0/280	1.21	2/427 (0.5%)
3	C	0.95	0/330	0.82	0/508
All	All	0.69	0/2748	0.75	2/3810 (0.1%)

There are no bond length outliers.

All (2) bond angle outliers are listed below:

Mol	Chain	Res	Type	Atoms	Z	Observed(^{o})	Ideal(^{o})
2	B	10	DT	N3-C4-O4	5.41	123.14	119.90
2	B	10	DT	C5-C4-O4	-5.29	121.20	124.90

There are no chirality outliers.

There are no planarity outliers.

5.2 Close contacts (i)

In the following table, the Non-H and H(model) columns list the number of non-hydrogen atoms and hydrogen atoms in the chain respectively. The H(added) column lists the number of hydrogens added by MolProbity. The Clashes column lists the number of clashes within the asymmetric unit, and the number in parentheses is this value normalized per 1000 atoms of the molecule in the chain. The Symm-Clashes column gives symmetry related clashes, in the same way as for the Clashes column.

Mol	Chain	Non-H	H(model)	H(added)	Clashes	Symm-Clashes
1	A	2101	0	0	3	0
2	B	266	0	0	0	0
3	C	291	0	0	0	0
4	A	11	0	0	0	0

Continued on next page...

Continued from previous page...

Mol	Chain	Non-H	H(model)	H(added)	Clashes	Symm-Clashes
5	A	6	0	8	1	0
6	A	50	0	0	1	0
6	B	5	0	0	0	0
6	C	3	0	0	0	0
All	All	2733	0	8	3	0

Clashscore is defined as the number of clashes calculated for the entry per 1000 atoms (including hydrogens) of the entry. The overall clashscore for this entry is 3.

All (3) close contacts within the same asymmetric unit are listed below.

Atom-1	Atom-2	Distance(Å)	Clash(Å)
1:A:187:LYS:NZ	1:A:271:LYS:O	2.40	0.54
1:A:74:ARG:NH2	5:A:302:GOL:H12	2.35	0.41
1:A:68:ARG:NH2	6:A:401:HOH:O	2.54	0.41

There are no symmetry-related clashes.

5.3 Torsion angles

5.3.1 Protein backbone (i)

In the following table, the Percentiles column shows the percent Ramachandran outliers of the chain as a percentile score with respect to all X-ray entries followed by that with respect to entries of similar resolution. The Analysed column shows the number of residues for which the backbone conformation was analysed, and the total number of residues.

Mol	Chain	Analysed	Favoured	Allowed	Outliers	Percentiles	
1	A	262/271 (97%)	256 (98%)	6 (2%)	0	100	100

There are no Ramachandran outliers to report.

5.3.2 Protein sidechains (i)

In the following table, the Percentiles column shows the percent sidechain outliers of the chain as a percentile score with respect to all X-ray entries followed by that with respect to entries of similar resolution. The Analysed column shows the number of residues for which the sidechain conformation was analysed, and the total number of residues.

Mol	Chain	Analysed	Rotameric	Outliers	Percentiles	
1	A	223/239 (93%)	222 (100%)	1 (0%)	95	98

All (1) residues with a non-rotameric sidechain are listed below:

Mol	Chain	Res	Type
1	A	159	TYR

Some sidechains can be flipped to improve hydrogen bonding and reduce clashes. There are no such sidechains identified.

5.3.3 RNA ⓘ

There are no RNA chains in this entry.

5.4 Non-standard residues in protein, DNA, RNA chains ⓘ

1 non-standard protein/DNA/RNA residue is modelled in this entry.

In the following table, the Counts columns list the number of bonds (or angles) for which Mogul statistics could be retrieved, the number of bonds (or angles) that are observed in the model and the number of bonds (or angles) that are defined in the chemical component dictionary. The Link column lists molecule types, if any, to which the group is linked. The Z score for a bond length (or angle) is the number of standard deviations the observed value is removed from the expected value. A bond length (or angle) with $|Z| > 2$ is considered an outlier worth inspection. RMSZ is the root-mean-square of all Z scores of the bond lengths (or angles).

Mol	Type	Chain	Res	Link	Bond lengths			Bond angles		
					Counts	RMSZ	#$\|Z\| > 2$	Counts	RMSZ	#$\|Z\| > 2$
2	3DR	B	7	2	9,11,12	3.24	5 (55%)	11,14,17	0.98	1 (9%)

In the following table, the Chirals column lists the number of chiral outliers, the number of chiral centers analysed, the number of these observed in the model and the number defined in the chemical component dictionary. Similar counts are reported in the Torsion and Rings columns. '-' means no outliers of that kind were identified.

Mol	Type	Chain	Res	Link	Chirals	Torsions	Rings
2	3DR	B	7	2	-	0/4/15/16	0/1/1/1

All (5) bond length outliers are listed below:

Mol	Chain	Res	Type	Atoms	Z	Observed(Å)	Ideal(Å)
2	B	7	3DR	C2'-C3'	-7.10	1.39	1.52
2	B	7	3DR	O5'-C5'	-4.92	1.37	1.44
2	B	7	3DR	O4'-C4'	-2.86	1.39	1.44
2	B	7	3DR	P-OP1	2.20	1.49	1.46

Continued on next page...

Continued from previous page...

Mol	Chain	Res	Type	Atoms	Z	Observed(Å)	Ideal(Å)
2	B	7	3DR	O3'-C3'	-2.03	1.38	1.43

All (1) bond angle outliers are listed below:

Mol	Chain	Res	Type	Atoms	Z	Observed(^{o})	Ideal(^{o})
2	B	7	3DR	O3'-C3'-C2'	-2.16	106.40	111.79

There are no chirality outliers.

There are no torsion outliers.

There are no ring outliers.

5.5 Carbohydrates (i)

There are no carbohydrates in this entry.

5.6 Ligand geometry (i)

2 ligands are modelled in this entry.

In the following table, the Counts columns list the number of bonds (or angles) for which Mogul statistics could be retrieved, the number of bonds (or angles) that are observed in the model and the number of bonds (or angles) that are defined in the chemical component dictionary. The Link column lists molecule types, if any, to which the group is linked. The Z score for a bond length (or angle) is the number of standard deviations the observed value is removed from the expected value. A bond length (or angle) with $|Z| > 2$ is considered an outlier worth inspection. RMSZ is the root-mean-square of all Z scores of the bond lengths (or angles).

Mol	Type	Chain	Res	Link	Bond lengths			Bond angles		
					Counts	RMSZ	$\#\|Z\| > 2$	Counts	RMSZ	$\#\|Z\| > 2$
4	2ON	A	301	1	12,12,12	1.07	0	14,17,17	4.90	5 (35%)
5	GOL	A	302	-	5,5,5	0.24	0	5,5,5	1.13	1 (20%)

In the following table, the Chirals column lists the number of chiral outliers, the number of chiral centers analysed, the number of these observed in the model and the number defined in the chemical component dictionary. Similar counts are reported in the Torsion and Rings columns. '-' means no outliers of that kind were identified.

Mol	Type	Chain	Res	Link	Chirals	Torsions	Rings
4	2ON	A	301	1	-	0/0/0/0	0/2/2/2
5	GOL	A	302	-	-	0/4/4/4	0/0/0/0

There are no bond length outliers.

All (6) bond angle outliers are listed below:

Mol	Chain	Res	Type	Atoms	Z	Observed(^{o})	Ideal(^{o})
4	A	301	2ON	C6-C5-N7	13.55	135.97	134.14
4	A	301	2ON	N9-C4-N3	9.49	132.86	124.53
4	A	301	2ON	C5-C4-N3	-4.94	120.38	126.07
4	A	301	2ON	C4-C5-N7	-4.04	106.00	109.93
4	A	301	2ON	C2-N3-C4	3.47	118.41	114.26
5	A	302	GOL	C3-C2-C1	-2.42	101.18	111.00

There are no chirality outliers.

There are no torsion outliers.

There are no ring outliers.

5.7 Other polymers (i)

There are no such residues in this entry.

5.8 Polymer linkage issues

There are no chain breaks in this entry.

6 Fit of model and data (i)

6.1 Protein, DNA and RNA chains (i)

In the following table, the column labelled '#RSRZ> 2' contains the number (and percentage) of RSRZ outliers, followed by percent RSRZ outliers for the chain as percentile scores relative to all X-ray entries and entries of similar resolution. The OWAB column contains the minimum, median, 95th percentile and maximum values of the occupancy-weighted average B-factor per residue. The column labelled 'Q< 0.9' lists the number of (and percentage) of residues with an average occupancy less than 0.9.

Mol	Chain	Analysed	<RSRZ>	#RSRZ>2			OWAB(Å^2)	Q<0.9
1	A	266/271 (98%)	-0.21	0	100	100	30, 45, 73, 118	0
2	B	14/14 (100%)	1.02	3 (21%)	1	1	35, 61, 94, 96	0
3	C	14/14 (100%)	0.66	2 (14%)	3	3	46, 73, 94, 99	0
All	All	294/299 (98%)	-0.11	5 (1%)	67	65	30, 46, 82, 118	0

All (5) RSRZ outliers are listed below:

Mol	Chain	Res	Type	RSRZ
2	B	14	DG	5.0
2	B	1	DC	4.1
3	C	15	DG	3.5
2	B	13	DC	2.7
3	C	28	DA	2.4

6.2 Non-standard residues in protein, DNA, RNA chains (i)

In the following table, the Atoms column lists the number of modelled atoms in the group and the number defined in the chemical component dictionary. LLDF column lists the quality of electron density of the group with respect to its neighbouring residues in protein, DNA or RNA chains. The B-factors column lists the minimum, median, 95th percentile and maximum values of B factors of atoms in the group. The column labelled 'Q< 0.9' lists the number of atoms with occupancy less than 0.9.

Mol	Type	Chain	Res	Atoms	RSR	LLDF	B-factors(Å^2)	Q<0.9
2	3DR	B	7	11/12	0.13	-0.04	37,44,49,51	0

6.3 Carbohydrates (i)

There are no carbohydrates in this entry.

6.4 Ligands (i)

In the following table, the Atoms column lists the number of modelled atoms in the group and the number defined in the chemical component dictionary. LLDF column lists the quality of electron density of the group with respect to its neighbouring residues in protein, DNA or RNA chains. The B-factors column lists the minimum, median, 95th percentile and maximum values of B factors of atoms in the group. The column labelled 'Q< 0.9' lists the number of atoms with occupancy less than 0.9.

Mol	Type	Chain	Res	Atoms	RSR	LLDF	B-factors($Å^2$)	Q<0.9
5	GOL	A	302	6/6	0.17	1.43	50,53,55,61	0
4	2ON	A	301	11/11	0.16	-0.19	74,84,91,134	0

6.5 Other polymers (i)

There are no such residues in this entry.

Full wwPDB X-ray Structure Validation Report (i)

Apr 28, 2014 – 04:22 PM EDT

PDB ID	:	4PDI
Title	:	Crystal structure of a complex between an inhibited LlFpg and a N7-Benzyl-Fapy-dG containing DNA
Authors	:	Coste, F.; Castaing, B.
Deposited on	:	2014-04-18
Resolution	:	2.10 Å(reported)

This is a full wwPDB validation report for a publicly released PDB entry.
We welcome your comments at validation@mail.wwpdb.org
A user guide is available at http://wwpdb.org/ValidationPDFNotes.html

The following versions of software and data (see references) were used in the production of this report:

MolProbity	:	4.02b-467
Mogul	:	1.16 November 2013
Xtriage (Phenix)	:	dev-1439
EDS	:	stable23106
Percentile statistics	:	21963
Refmac	:	5.8.0049
CCP4	:	6.3.0 (Settle)
Ideal geometry (proteins)	:	Engh & Huber (2001)
Ideal geometry (DNA, RNA)	:	Parkinson et. al. (1996)
Validation Pipeline (wwPDB-VP)	:	stable23106

1 Overall quality at a glance (i)

The reported resolution of this entry is 2.10 Å.

Percentile scores (ranging between 0-100) for global validation metrics of the entry are shown in the following graphic. The table shows the number of entries on which the scores are based.

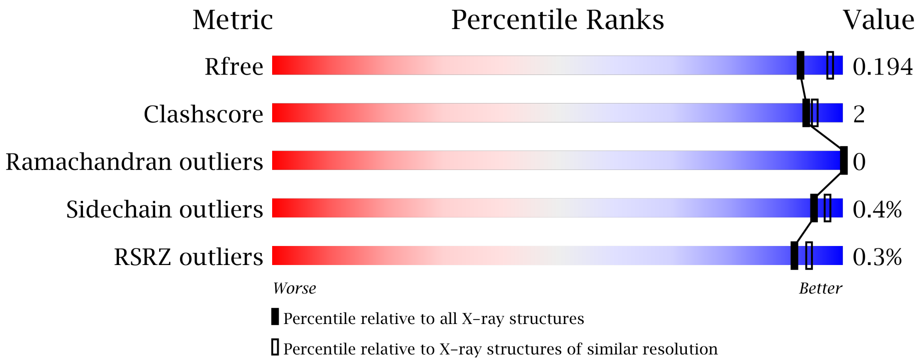

Metric	Whole archive (#Entries)	Similar resolution (#Entries, resolution range(Å))
R_{free}	66092	3012 (2.10-2.10)
Clashscore	79885	3649 (2.10-2.10)
Ramachandran outliers	78287	3610 (2.10-2.10)
Sidechain outliers	78261	3611 (2.10-2.10)
RSRZ outliers	66119	3013 (2.10-2.10)

The table below summarises the geometric issues observed across the polymeric chains and their fit to the electron density. The red, orange, yellow and green segments on the lower bar indicate the fraction of residues that contain outliers for >=3, 2, 1 and 0 types of geometric quality criteria. The upper red bar (where present) indicates the fraction of residues that have poor fit to the electron density.

Mol	Chain	Length	Quality of chain
1	A	271	
2	B	14	
3	C	14	

The following table lists non-polymeric compounds that are outliers for geometric or electron-density-fit criteria:

Mol	Type	Chain	Res	Geometry	Electron density
4	2ON	A	300	-	X

2 Entry composition (i)

There are 5 unique types of molecules in this entry. The entry contains 3014 atoms, of which 0 are hydrogen and 0 are deuterium.

In the tables below, the ZeroOcc column contains the number of atoms modelled with zero occupancy, the AltConf column contains the number of residues with at least one atom in alternate conformation and the Trace column contains the number of residues modelled with at most 2 atoms.

- Molecule 1 is a protein called Formamidopyrimidine-DNAglycosylase.

Mol	Chain	Residues	Atoms					ZeroOcc	AltConf	Trace
			Total	C	N	O	S			
1	A	268	2138	1373	360	397	8	0	3	0

- Molecule 2 is a DNA chain called DNA (5'-D(*CP*TP*CP*TP*TP*TP*(SOS)P*TP*TP*TP*CP*TP*CP*G)-3').

Mol	Chain	Residues	Atoms					ZeroOcc	AltConf	Trace
			Total	C	N	O	P			
2	B	14	285	144	38	90	13	0	0	0

- Molecule 3 is a DNA chain called DNA (5'-D(*GP*CP*GP*AP*GP*AP*AP*AP*CP*AP*AP*AP*GP*A)-3').

Mol	Chain	Residues	Atoms					ZeroOcc	AltConf	Trace
			Total	C	N	O	P			
3	C	14	291	138	66	74	13	0	0	0

- Molecule 4 is 2-sulfanyl-1,9-dihydro-6H-purin-6-one (three-letter code: 2ON) (formula: $C_5H_4N_4OS$).

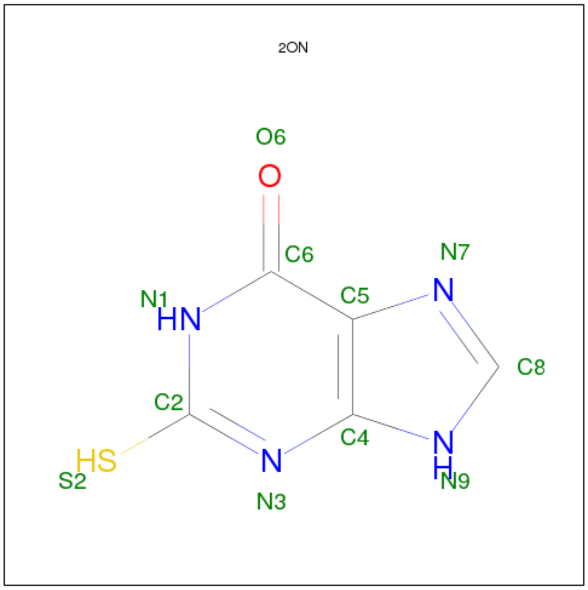

Mol	Chain	Residues	Atoms	ZeroOcc	AltConf
4	A	1	Total C N O S<br>11 5 4 1 1	0	0

- Molecule 5 is water.

Mol	Chain	Residues	Atoms	ZeroOcc	AltConf
5	A	257	Total O<br>257 257	0	0
5	B	21	Total O<br>21 21	0	0
5	C	11	Total O<br>11 11	0	0

3 Residue-property plots (i)

These plots are drawn for all protein, RNA and DNA chains in the entry. The first graphic for a chain summarises the proportions of errors displayed in the second graphic. The second graphic shows the sequence view annotated by issues in geometry and electron density. Residues are color-coded according to the number of geometric quality criteria for which they contain at least one outlier: green = 0, yellow = 1, orange = 2 and red = 3 or more. A red dot above a residue indicates a poor fit to the electron density (RSRZ > 2). Stretches of 2 or more consecutive residues without any outlier are shown as a green connector. Residues present in the sample, but not in the model, are shown in grey.

- Molecule 1: Formamidopyrimidine-DNAglycosylase

Chain A:

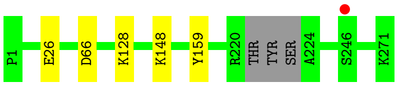

- Molecule 2: DNA (5'-D(*CP*TP*CP*TP*TP*TP*(SOS)P*TP*TP*TP*CP*TP*CP*G)-3')

Chain B:

There are no outlier residues recorded for this chain.

- Molecule 3: DNA (5'-D(*GP*CP*GP*AP*GP*AP*AP*AP*CP*AP*AP*AP*GP*A)-3')

Chain C:

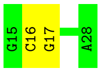

4 Data and refinement statistics

Property	Value	Source
Space group	P 41 21 2	Depositor
Cell constants<br>a, b, c, α, β, γ	91.53Å 91.53Å 140.97Å<br>90.00° 90.00° 90.00°	Depositor
Resolution (Å)	47.67 – 2.10<br>47.67 – 2.10	Depositor<br>EDS
% Data completeness<br>(in resolution range)	100.0 (47.67-2.10)<br>100.0 (47.67-2.10)	Depositor<br>EDS
R_{merge}	0.10	Depositor
R_{sym}	(Not available)	Depositor
$< I/\sigma(I) >$ [1]	2.09 (at 2.10Å)	Xtriage
Refinement program	PHENIX (phenix.refine: 1.8.4_1496)	Depositor
R, R_{free}	0.160 , 0.190<br>0.164 , 0.194	Depositor<br>DCC
R_{free} test set	1783 reflections (5.01%)	DCC
Wilson B-factor (Å^2)	33.4	Xtriage
Anisotropy	0.032	Xtriage
Bulk solvent k_{sol}(e/Å^3), B_{sol}(Å^2)	0.37 , 53.0	EDS
Estimated twinning fraction	No twinning to report.	Xtriage
L-test for twinning	$< \|L\| > = 0.49$, $< L^2 > = 0.33$	Xtriage
Outliers	0 of 35597 reflections	Xtriage
F_o,F_c correlation	0.95	EDS
Total number of atoms	3014	wwPDB-VP
Average B, all atoms (Å^2)	39.0	wwPDB-VP

Xtriage's analysis on translational NCS is as follows: *The largest off-origin peak in the Patterson function is 3.66% of the height of the origin peak. No significant pseudotranslation is detected.*

[1]Intensities estimated from amplitudes.

5 Model quality (i)

5.1 Standard geometry (i)

Bond lengths and bond angles in the following residue types are not validated in this section: SOS, 2ON

The Z score for a bond length (or angle) is the number of standard deviations the observed value is removed from the expected value. A bond length (or angle) with $|Z| > 5$ is considered an outlier worth inspection. RMSZ is the root-mean-square of all Z scores of the bond lengths (or angles).

Mol	Chain	Bond lengths		Bond angles	
		RMSZ	$\#\|Z\| > 5$	RMSZ	$\#\|Z\| > 5$
1	A	0.40	0/2184	0.53	0/2936
2	B	0.80	0/280	1.18	0/427
3	C	0.70	0/330	0.74	0/508
All	All	0.50	0/2794	0.67	0/3871

There are no bond length outliers.

There are no bond angle outliers.

There are no chirality outliers.

There are no planarity outliers.

5.2 Close contacts (i)

In the following table, the Non-H and H(model) columns list the number of non-hydrogen atoms and hydrogen atoms in the chain respectively. The H(added) column lists the number of hydrogens added by MolProbity. The Clashes column lists the number of clashes within the asymmetric unit, and the number in parentheses is this value normalized per 1000 atoms of the molecule in the chain. The Symm-Clashes column gives symmetry related clashes, in the same way as for the Clashes column.

Mol	Chain	Non-H	H(model)	H(added)	Clashes	Symm-Clashes
1	A	2138	0	0	4	0
2	B	285	0	174	0	0
3	C	291	0	156	1	0
4	A	11	0	0	0	0
5	A	257	0	0	3	0
5	B	21	0	0	0	0
5	C	11	0	0	0	0
All	All	3014	0	330	5	0

Clashscore is defined as the number of clashes calculated for the entry per 1000 atoms (including hydrogens) of the entry. The overall clashscore for this entry is 2.

All (5) close contacts within the same asymmetric unit are listed below.

Atom-1	Atom-2	Distance(Å)	Clash(Å)
1:A:148:LYS:NZ	5:A:536:HOH:O	2.32	0.62
1:A:26:GLU:OE2	5:A:531:HOH:O	2.17	0.56
3:C:16:DC:H2'	3:C:17:DG:C8	2.42	0.54
1:A:128:LYS:NZ	5:A:401:HOH:O	2.43	0.51
1:A:66:ASP:N	1:A:66:ASP:OD1	2.54	0.41

There are no symmetry-related clashes.

5.3 Torsion angles

5.3.1 Protein backbone (i)

In the following table, the Percentiles column shows the percent Ramachandran outliers of the chain as a percentile score with respect to all X-ray entries followed by that with respect to entries of similar resolution. The Analysed column shows the number of residues for which the backbone conformation was analysed, and the total number of residues.

Mol	Chain	Analysed	Favoured	Allowed	Outliers	Percentiles
1	A	267/271 (98%)	260 (97%)	7 (3%)	0	100 100

There are no Ramachandran outliers to report.

5.3.2 Protein sidechains (i)

In the following table, the Percentiles column shows the percent sidechain outliers of the chain as a percentile score with respect to all X-ray entries followed by that with respect to entries of similar resolution. The Analysed column shows the number of residues for which the sidechain conformation was analysed, and the total number of residues.

Mol	Chain	Analysed	Rotameric	Outliers	Percentiles
1	A	227/239 (95%)	226 (100%)	1 (0%)	95 97

All (1) residues with a non-rotameric sidechain are listed below:

Mol	Chain	Res	Type
1	A	159	TYR

Some sidechains can be flipped to improve hydrogen bonding and reduce clashes. There are no such sidechains identified.

5.3.3 RNA (i)

There are no RNA chains in this entry.

5.4 Non-standard residues in protein, DNA, RNA chains (i)

1 non-standard protein/DNA/RNA residue is modelled in this entry.

In the following table, the Counts columns list the number of bonds (or angles) for which Mogul statistics could be retrieved, the number of bonds (or angles) that are observed in the model and the number of bonds (or angles) that are defined in the chemical component dictionary. The Link column lists molecule types, if any, to which the group is linked. The Z score for a bond length (or angle) is the number of standard deviations the observed value is removed from the expected value. A bond length (or angle) with $|Z| > 2$ is considered an outlier worth inspection. RMSZ is the root-mean-square of all Z scores of the bond lengths (or angles).

Mol	Type	Chain	Res	Link	Bond lengths			Bond angles		
					Counts	RMSZ	#|Z| > 2	Counts	RMSZ	#|Z| > 2
2	SOS	B	7	2	30,32,33	2.36	6 (20%)	39,44,47	2.07	10 (25%)

In the following table, the Chirals column lists the number of chiral outliers, the number of chiral centers analysed, the number of these observed in the model and the number defined in the chemical component dictionary. Similar counts are reported in the Torsion and Rings columns. '-' means no outliers of that kind were identified.

Mol	Type	Chain	Res	Link	Chirals	Torsions	Rings
2	SOS	B	7	2	-	0/18/31/32	0/3/3/3

All (6) bond length outliers are listed below:

Mol	Chain	Res	Type	Atoms	Z	Observed(Å)	Ideal(Å)
2	B	7	SOS	O5'-C5'	-5.53	1.36	1.44
2	B	7	SOS	C2-N2	5.29	1.40	1.32
2	B	7	SOS	C6-N1	4.81	1.43	1.36
2	B	7	SOS	C4-N3	4.66	1.43	1.34
2	B	7	SOS	P-OP1	4.08	1.51	1.46
2	B	7	SOS	C6'-C1'	4.01	1.59	1.53

All (10) bond angle outliers are listed below:

Mol	Chain	Res	Type	Atoms	Z	Observed(^{o})	Ideal(^{o})
2	B	7	SOS	C6-C5-C4	8.06	120.84	114.43
2	B	7	SOS	C6-C5-N7	-4.13	116.62	119.75
2	B	7	SOS	C4-N9-C1'	-3.70	119.13	123.86
2	B	7	SOS	C5-N7-C8	-3.00	116.92	120.50
2	B	7	SOS	C9-N7-C8	2.82	124.76	120.00
2	B	7	SOS	C3'-C2'-C1'	-2.66	101.82	104.59
2	B	7	SOS	P-O5'-C5'	-2.35	113.72	122.98
2	B	7	SOS	O8-C8-N7	-2.21	121.00	124.57
2	B	7	SOS	N1-C2-N3	2.19	124.74	121.78
2	B	7	SOS	C5-C4-N9	-2.01	121.59	123.55

There are no chirality outliers.

There are no torsion outliers.

There are no ring outliers.

5.5 Carbohydrates (i)

There are no carbohydrates in this entry.

5.6 Ligand geometry (i)

1 ligand is modelled in this entry.

In the following table, the Counts columns list the number of bonds (or angles) for which Mogul statistics could be retrieved, the number of bonds (or angles) that are observed in the model and the number of bonds (or angles) that are defined in the chemical component dictionary. The Link column lists molecule types, if any, to which the group is linked. The Z score for a bond length (or angle) is the number of standard deviations the observed value is removed from the expected value. A bond length (or angle) with $|Z| > 2$ is considered an outlier worth inspection. RMSZ is the root-mean-square of all Z scores of the bond lengths (or angles).

Mol	Type	Chain	Res	Link	Bond lengths			Bond angles		
					Counts	RMSZ	$\#\|Z\| > 2$	Counts	RMSZ	$\#\|Z\| > 2$
4	2ON	A	300	1	12,12,12	3.65	5 (41%)	14,17,17	4.24	6 (42%)

In the following table, the Chirals column lists the number of chiral outliers, the number of chiral centers analysed, the number of these observed in the model and the number defined in the chemical component dictionary. Similar counts are reported in the Torsion and Rings columns. '-' means no outliers of that kind were identified.

Mol	Type	Chain	Res	Link	Chirals	Torsions	Rings
4	2ON	A	300	1	-	0/0/0/0	0/2/2/2

All (5) bond length outliers are listed below:

Mol	Chain	Res	Type	Atoms	Z	Observed(Å)	Ideal(Å)
4	A	300	2ON	C4-N3	9.75	1.44	1.34
4	A	300	2ON	C2-N3	4.87	1.44	1.33
4	A	300	2ON	C6-N1	3.84	1.42	1.36
4	A	300	2ON	C8-N9	3.61	1.38	1.34
4	A	300	2ON	C2-N1	2.58	1.43	1.35

All (6) bond angle outliers are listed below:

Mol	Chain	Res	Type	Atoms	Z	Observed(^{o})	Ideal(^{o})
4	A	300	2ON	C6-C5-N7	-9.90	132.81	134.14
4	A	300	2ON	N9-C4-N3	8.54	132.03	124.53
4	A	300	2ON	C2-N3-C4	5.48	120.80	114.26
4	A	300	2ON	C5-C4-N3	-5.35	119.91	126.07
4	A	300	2ON	C4-C5-N7	-2.57	107.43	109.93
4	A	300	2ON	C5-C4-N9	2.03	108.06	106.07

There are no chirality outliers.

There are no torsion outliers.

There are no ring outliers.

5.7 Other polymers (i)

There are no such residues in this entry.

5.8 Polymer linkage issues

There are no chain breaks in this entry.

6 Fit of model and data (i)

6.1 Protein, DNA and RNA chains (i)

In the following table, the column labelled '#RSRZ> 2' contains the number (and percentage) of RSRZ outliers, followed by percent RSRZ outliers for the chain as percentile scores relative to all X-ray entries and entries of similar resolution. The OWAB column contains the minimum, median, 95th percentile and maximum values of the occupancy-weighted average B-factor per residue. The column labelled 'Q< 0.9' lists the number of (and percentage) of residues with an average occupancy less than 0.9.

Mol	Chain	Analysed	<RSRZ>	#RSRZ>2			OWAB(Å^2)	Q<0.9
1	A	268/271 (98%)	-0.69	1 (0%)	90	92	23, 33, 51, 100	3 (1%)
2	B	13/14 (92%)	0.07	0	100	100	29, 47, 60, 65	0
3	C	14/14 (100%)	-0.16	0	100	100	32, 57, 65, 72	0
All	All	295/299 (98%)	-0.63	1 (0%)	91	94	23, 33, 60, 100	3 (1%)

All (1) RSRZ outliers are listed below:

Mol	Chain	Res	Type	RSRZ
1	A	246	SER	2.2

6.2 Non-standard residues in protein, DNA, RNA chains (i)

In the following table, the Atoms column lists the number of modelled atoms in the group and the number defined in the chemical component dictionary. LLDF column lists the quality of electron density of the group with respect to its neighbouring residues in protein, DNA or RNA chains. The B-factors column lists the minimum, median, 95th percentile and maximum values of B factors of atoms in the group. The column labelled 'Q< 0.9' lists the number of atoms with occupancy less than 0.9.

Mol	Type	Chain	Res	Atoms	RSR	LLDF	B-factors(Å^2)	Q<0.9
2	SOS	B	7	30/31	0.09	0.83	23,28,35,37	0

6.3 Carbohydrates (i)

There are no carbohydrates in this entry.

6.4 Ligands (i)

In the following table, the Atoms column lists the number of modelled atoms in the group and the number defined in the chemical component dictionary. LLDF column lists the quality of electron density of the group with respect to its neighbouring residues in protein, DNA or RNA chains. The B-factors column lists the minimum, median, 95th percentile and maximum values of B factors of atoms in the group. The column labelled 'Q< 0.9' lists the number of atoms with occupancy less than 0.9.

Mol	Type	Chain	Res	Atoms	RSR	LLDF	B-factors($Å^2$)	Q<0.9
4	2ON	A	300	11/11	0.17	3.45	66,91,99,101	0

6.5 Other polymers (i)

There are no such residues in this entry.
